# When Salt Meddles Between Plant, Soil, and Microorganisms

**DOI:** 10.3389/fpls.2020.553087

**Published:** 2020-09-16

**Authors:** Anna Otlewska, Melania Migliore, Katarzyna Dybka-Stępień, Andrea Manfredini, Katarzyna Struszczyk-Świta, Rosario Napoli, Aneta Białkowska, Loredana Canfora, Flavia Pinzari

**Affiliations:** ^1^ Faculty of Biotechnology and Food Sciences, Institute of Fermentation Technology and Microbiology, Lodz University of Technology, Lodz, Poland; ^2^ Research Centre for Agriculture and Environment, Council for Agricultural Research and Economics, Rome, Italy; ^3^ Faculty of Biotechnology and Food Sciences, Institute of Molecular and Industrial Biotechnology, Lodz University of Technology, Lodz, Poland; ^4^ Institute for Biological Systems, Council of National Research of Italy (CNR), Rome, Italy

**Keywords:** ****extreme environment, halophilic microorganisms, endophytes, PGPR—plant growth-promoting rhizobacteria, agriculture, microbial metabolism, salty soils

## Abstract

In extreme environments, the relationships between species are often exclusive and based on complex mechanisms. This review aims to give an overview of the microbial ecology of saline soils, but in particular of what is known about the interaction between plants and their soil microbiome, and the mechanisms linked to higher resistance of some plants to harsh saline soil conditions. Agricultural soils affected by salinity is a matter of concern in many countries. Soil salinization is caused by readily soluble salts containing anions like chloride, sulphate and nitrate, as well as sodium and potassium cations. Salinity harms plants because it affects their photosynthesis, respiration, distribution of assimilates and causes wilting, drying, and death of entire organs. Despite these life-unfavorable conditions, saline soils are unique ecological niches inhabited by extremophilic microorganisms that have specific adaptation strategies. Important traits related to the resistance to salinity are also associated with the rhizosphere-microbiota and the endophytic compartments of plants. For some years now, there have been studies dedicated to the isolation and characterization of species of plants’ endophytes living in extreme environments. The metabolic and biotechnological potential of some of these microorganisms is promising. However, the selection of microorganisms capable of living in association with host plants and promoting their survival under stressful conditions is only just beginning. Understanding the mechanisms of these processes and the specificity of such interactions will allow us to focus our efforts on species that can potentially be used as beneficial bioinoculants for crops.

## Introduction

Soil salinization is a process of localised accumulation of soluble salts. This phenomenon today is unanimously considered a severe threat to agricultural lands as it directly undermines the value and quality of soil (Ammari et al., 2013; Daliakopoulos et al., 2016). The soil is a complex system in continuous evolution and dynamic equilibrium with the other environmental components, sensitive to the effects of climate change and human activities (Smith et al., 2012). Still, it constitutes a substantially non-renewable resource in the sense that the rate of its degradation is potentially rapid (Zewdu et al., 2017), while the soil formation and regeneration processes are extremely slow.

Globally it has been estimated that 33% of the irrigated agricultural land and over 20% of the total cultivated land of the world is saline. If the current trend of salinization is maintained, then by 2050 we will see an approximately 30% increase in the cultivated land salinity. This means that agricultural productivity will be reduced due to the decline in cultivable land, with a consequent increase in the number of people suffering from hunger. Plants are the first in the chain of food production to be hit by salinity stress, which hampers their basic physiological and biochemical processes, such as water absorption and photosynthesis, thus resulting in overall reduced growth (Vaishnav et al., 2016). However, plants develop various morphological, physiological, biochemical, and molecular strategies in response to salinity in their environment (Meng et al., 2018).

In recent decades, significant overviews about mechanisms of salinity tolerance in plants have been shared by several authors (Munns and Tester, 2008; Deinlein et al., 2014; Gupta and Huang, 2014; Meng et al., 2018). In general, two types of plant adaptation to high salt concentration in the soil are distinguished: avoidance and tolerance strategy (Dajic, 2006). The first consisting of creating physical, physiological or metabolic barriers that counteract the penetration of stress factors in the plant, which causes morphological and physiological changes at the whole plant level. The tolerance strategy is based instead on plant ability to survive in stress conditions through cellular, molecular, and biochemical modifications aimed at minimising stress effects (Bahmani et al., 2015). Interestingly, those plants have a strong influence on shaping the rhizosphere and endorhiza microbiome (Abedinzadeh et al., 2019).

In particular, some plant growth-promoting rhizobacteria (PGPR) may exert a direct stimulation on plants’ growth and development by providing them with fixed nitrogen, phytohormones (IAA, GB), iron (Egamberdieva et al., 2019), and soluble phosphate (Shrivastava and Kumar, 2015) that help to overcome the effects of salinity stress. Another mechanism that minimizes the impact of salinity consists in the production of substances (proline, trehalose, glycine, betaine) that work as osmoprotectant for plants’ cells (Kushwaha et al., 2019). Moreover, under various environmental stresses, plants typically produce ethylene, from the precursor 1-aminocyclopropane-1-carboxylic acid (ACC). It is a hormone that limits plants’ growth in conditions of high salinity, high temperatures, drought, or at the presence of toxic metals or organic pollutants. PGPRs produce a particular enzyme that degrades ethylene (ACC-deaminase), allowing the plants’ roots to develop despite the environmental stress (Glick, 2012).

When salt meddles between soil, plant and microorganisms in and around the rhizosphere, a unique extreme environment is created, that provides a scene for mutualistic relationships. In this system, plant-endophytes and plant-microbiome interactions play a crucial role in the activation or stimulation of different adaptation mechanisms for survival in saline soil (Acosta-Motos et al., 2017). In this review, we provide information about natural and anthropic causes of soil salinity and discuss the plant’s strategies for stress management. An overview of the known interactions occurring between endophytic communities and host plants is provided along with a discussion on the potential of plant growth-promoting rhizobacteria (PGPR) for increasing plant’s salt stress tolerance.

## Natural and Anthropic Causes of Soil Salinity Affecting Agricultural Land Use

All soils naturally contain a mixture of salts soluble in water, and some of these are essential for plant development. Their origin is mainly from the meteoric alteration of the igneous rocks of the lithosphere. Following the hydrological and climatic events, in past ages, there has been the deposition of large quantities of salts in sedimentary rocks, in surface and underground waters, in seas and oceans (Daliakopoulos et al., 2016; Zaman et al., 2018). From these deposits, through various mechanisms, the salts reach the soil. In particular conditions, the formation of a water evaporation front in the soil moves water by evaporation rather than by percolation, so the salts remain in the soil and accumulate (Datta and de Jong, 2002).

Soil salinity can be distinguished in primary and secondary. The first depends on factors related mostly to the lithology of the substrate (in particular hydrological characteristics), morphological characteristics of the area, intrinsic chemistry of soils and climatic factors (Schofield and Kirkby, 2003). If soil parent rock contains carbonate minerals or feldspar, physical or chemical weathering operated by water bring salts in solution, increasing their concentration in groundwater and consequently on the wetted topsoil layer. Soil porosity, texture and mineral composition influence soil hydrological properties which also depend on the salts’ accumulation on the soil surface. The amount of saline precipitates is in turn modulated by soil transpiration and the extent and characteristics of the capillary fringe. This kind of accumulation process is reported in different European areas (Schofield and Kirkby, 2003; Kováčová and Velísková, 2012; Gkiougkis et al., 2015; Daliakopoulos et al., 2016).

Another group of naturally saline soils originate from salty marine seawater in the coastal regions. This phenomenon can occur under short- or long-term periods. In short term periods, soil salinization can be caused by stochastic rapid events like floods or tsunamis that cause salinization on the surface of the geographical area beaten by the waves (Central Water Commission, 2017). In contrast, in a geological time scale, prolonged high tides can cause the formation of marine deposits. Soils of the coastal areas can be therefore easily affected by salinization, due to the intrusion of the marine seawater wedge inside the fresh groundwater (Daliakopoulos et al., 2016). Despite the saltwater in a natural environment usually stays below the fresh, because of its higher density, in many cases the water extraction by wells for different human uses could interfere by breaking this balance, inducing the recall of the saline wedge with rising saltwater and the consequent formation of an area of brackish water near the ground surface. This anthropic cause is mainly due to inadequate agricultural water management practices and could induce “secondary soil salinization” along time by increasing the salt content in the irrigation waters (Napoli and Vanino, 2011; Greggio et al., 2012).

Agriculture plays an essential role as one of the leading causes of secondary salinization given by the use of poor-quality water, often worsened by the presence of soil components that limit the leaching of salts, such as the presence of impermeable horizons and an unfavorable physiographic position.

Secondary salinity could also depend by the overuse of fertilizers, insecticides and fungicides, unsustainable use of the land, excessive drainage of the water tables (Datta and de Jong, 2002).

Salinization caused by overuse of fertilizers is due to different phenomena. The excessive use of nitrogen fertilizers both chemicals or organic can cause a rise in nitrate content in the soil that can be detected as a rise in Electrical Conductivity (EC) or interpreted through signs of crop disease often associated with high soil salinity (Gkiougkis et al., 2015). The use of fertilizers with a high content of potassium and sodium can cause soil degradation by the accumulation of these salts in particular conditions (Chang et al., 2018). The application of incorrect irrigation practices added to an excess of fertilization, together with particular climatic conditions sometimes may favor the accumulation of salts (Daliakopoulos et al., 2016). Poorly permeable silty-clay soils in climatic conditions with limited rainfall and high temperatures favor high evapotranspiration and therefore the quick accumulation of salts in the first layers of the soil (Chang et al., 2018)

It should be considered, however, that even in areas irrigated using “good” quality water, moderate levels of salinity were detected as a consequence of the irrigation methods applied and local arid climatic conditions. Vice versa, the phenomenon of salinization may not occur on lands irrigated for several years with waters rich in salts. These examples indicate that each area is characterized by different and peculiar balances, which influence its possible evolution. In certain soils, drained and with particular thermo-pluviometric trends, the accumulation of salts in the soils could be only temporary. A balance could quickly be established between accumulation and leaching that would allow over time, and with due care, to maintain agricultural activity (Machado and Serralheiro, 2017).

Secondary soil salinization caused by incorrect irrigation strategies is also associated with the use of wastewaters. When the use of poor quality water exceeds the natural buffering effect of the soil, a whole series of substances, such as insecticides and fungicides remain on the ground which, as a result, cause an increase in salinity (Kováčová and Velísková, 2012; Rodríguez-Liébana et al., 2014). Another common cause of secondary salinization phenomenon is the consequence of the replacement of spontaneous vegetation (polyannual and primarily arboreal species) with crops (annual and exclusively herbaceous), characterized by superficial, less deep root systems than the pristine vegetation. This artificial change of vegetation causes a drastic modification of the delicate hydrological balance. In essence, the reduced root systems of the new plant species, requiring less water than the original tall trees leads, over time, to a progressive rise of the water table and lower solubilization of the salts in the subsoil, causing them to rise together with the water until it affects the layer occupied by the roots of the crops (Bui, 2013). The water absorption from the roots and the evapotranspiration process inevitably causes a gradual accumulation of the salts on the surface, making the soil progressively inhospitable to agricultural plants and unsuitable for agriculture (Hanson et al., 1993; Machado and Serralheiro, 2017). The saline sources in secondary salinization could also come from the use of soil improvers that are themselves saline (i.e. gypsum or elemental sulphur), from manure and chemical fertilizers (Wallender and Tanji, 2012).

### Classification and Extension of Saline Soils

As reported by Bui in 2013, the definition of saline soil is confusing. Are considered saline the soils in which salt concentration can interfere with the capability of plants to absorb water, affecting their growth or more specifically a soil with an electric conductivity (EC) on a saturated soil paste extract >4 dS m^-1^ (Bui, 2013; Shrivastava and Kumar, 2015; Zaman et al., 2018). Salts concentration and the osmotic pressure of a saline soil also depend on soil texture and relative water characteristics more than only on the salt content (Darwish et al., 2005; Daliakopoulos et al., 2016; Zaman et al., 2018).

In literature, there are two most accepted classifications for saline soil. One is the *US Salinity Laboratory Staff Classiﬁcation* in which it is most commonly used the term “salt-affected soil” to indicate saline, saline-sodic and sodic soils. In this classification, a saline soil has an EC ≥4 dS m^-1^, exchangeable sodium percentage (ESP) <15 and pH <8.5 while saline-sodic differs only for ESP ≥15 and pH ≥8.5. Sodic soils have EC <4 dS m^-1^, ESP ≥15 and pH >8.5. These soils are also characterized by the loss of permeability to water caused by the disruption of soil aggregate operated mostly by the Na^+^ ions (Daliakopoulos et al., 2016; Machado and Serralheiro, 2017; Zaman et al., 2018) and the ensuing collapse of soil structure in the Natric Horizon.

According to the classification approach of *World Reference Base Classiﬁcation* (IUSS Working Group WRB, 2014), salt-affected soils are divided into two classes: solonchacks (saline soils with ECe >15 dSm^-1^ in the top 125 cm) and solonetz (are sodium-rich soils with an ESP > 15), both divided into subclasses (Zaman et al., 2018).

The Reference Group of Solonchacks is quite widespread in all the arid and semi-arid areas of North Africa, Near East, Central Asia, India, Iran and Iraq, Australia and the Americas. The Reference Group of Solonetz, mainly located in the steppe climatic regimes and flat landscapes with poor drainage, were mapped in Ukraine, Russian Federation, Eastern Europe, China, India, USA, Canada, Southern and Eastern Africa and Australia. Last studies and maps at world level reported an estimated area of about 260 Mha and 135 Mha covered by Solonchacks and Solonetz, respectively (Cherlet et al., 2018).

Apart from mapping the soils classified as “saline”, the exact extension and localisation of salinity based on its causes were hardly addressed geographically. Primary (natural) salinization was estimated to be slightly under 1 billion ha worldwide, secondary (artificially induced by human activities) occurs on around 77 Mha, mostly in intensively cultivated and irrigated areas of India, Pakistan, China, Iraq, and Iran. Because of the human activities linked to wrong water management, large areas of the Mediterranean Basin, Australia, Central Asia, the Middle East and Northern Africa are interested in the risk of salinization (Cherlet et al., 2018).

In Europe, secondary salinization affects approximately 4 Mha of European soils (Daliakopoulos et al., 2016) and focusing on the Mediterranean region. Soil salinization affects 25% of irrigated agricultural land. Along the Mediterranean coasts, soil salinity is the primary cause of desertification due to human activities. Artificially induced salinization caused by irrigated agriculture is affecting significant parts of southern Italy (Napoli and Vanino, 2011), Spain (e.g. the Ebro Valley), Hungary (e.g. Great Alfold), Greece, Cyprus, Portugal, France (West coast), the Dalmatian coast, Slovakia and Romania. Besides, North Europe countries (e.g. Denmark, Poland, Latvia, and Estonia) are facing similar issues (Daliakopoulos et al., 2016).

### Limitation to Agricultural Uses

Salinization represents a critical threat for agriculture because it can cause the alteration of the delicate balance of ecological processes occurring in soil.

The phenomenon of salinization represents only one of the multiple aspects that the accumulation of salts in soil causes (Shrivastava and Kumar, 2015; Nouri et al., 2017). The loss of soil occurs in terms of both surface subtraction (Peng et al., 2019), and the alteration of its chemical characteristics Nouri et al., 2017). The salinized soil readily undergoes to a further series of degradation processes, such as erosion (Ishida et al., 2009), a decrease of organic matter content (de Souza and Fay, 2014), local or widespread contamination (Hanson et al., 1993), sealing, compaction, and decline of biodiversity (Nouri et al., 2017). These processes contribute to compromising the quality of the soil and its ability to interact with the ecosystem to maintain biological productivity, environmental quality and promote the health of all living organisms (Salvati and Ferrara, 2015; Zaman et al., 2018).

Saline and sodic soils reduce the value and productivity of large areas around the world (Qadir et al., 2014; Acuña-Rodríguez et al., 2019). It is estimated that every day in the world 2000 hectares of arable land are lost due to salinization, and this problem can cause loss of yield for many crops of 10-25% and in exceptional conditions can lead to desertification (Zaman et al., 2018).

Geographical areas affected by salinity problems have an impact on both the environmental and socioeconomic sphere. These areas face the loss of productivity (Datta and de Jong, 2002) with a different degree of magnitude depending on the crops (Qadir et al., 2014) seeing their farmers sometimes reduced to poverty or forced to migrate searching for a new source of income (Ammari et al., 2013; Salvati and Ferrara, 2015; Zaman et al., 2018). Soil can become saline at first, causing a decrease in agricultural yield and then, in the long run, it can progressively turn into completely sterile (desertification) (Gorji et al., 2017; Cuevas et al., 2019).

The pollution from chemicals causes the water that returns to the water cycle to be of poor quality with negative consequences on agriculture and health, giving rise to a vicious cycle Zewdu et al., 2017). Soil salinization has a significant impact on the environment, causing landscape and consequently, ecosystem fragmentation (Cramer and Hobbs, 2002). A poor vegetative growth leads to the reduction of the protective role of the plants cover, enhancing soil degradation and erosion. The presence of chemical residues on the soil surface, together with salts, cause the elevation of toxic clouds of dust (Zewdu et al., 2017).

The enrichment of the soil with organic matter, humic substances, the application of biofertilizer containing microorganism and the application of fertilizers through irrigation water (fertigation) could help to alleviate the negative effect of salt accumulation on salt-sensitive crops (Darwish et al., 2005).

Given the importance of agricultural production, it is crucial to understand the impacts of salinization on different crops and to find effective strategies to reduce economic losses in salt-affected areas.

## Effects of Salinity Stress on Plant Growth

Soil salinity affects (directly or indirectly) both growth and reproduction of plants as a consequence of complex interactions between physicochemical properties of soil (salt content, poor aeration, an increase of crusting, hard setting, reduced infiltration, reduction of water uptake, and difficult root penetration) and plants’ morphological and physiological features (Rogers et al., 2005; Akbarimoghaddam et al., 2011).

**Figure 1:**
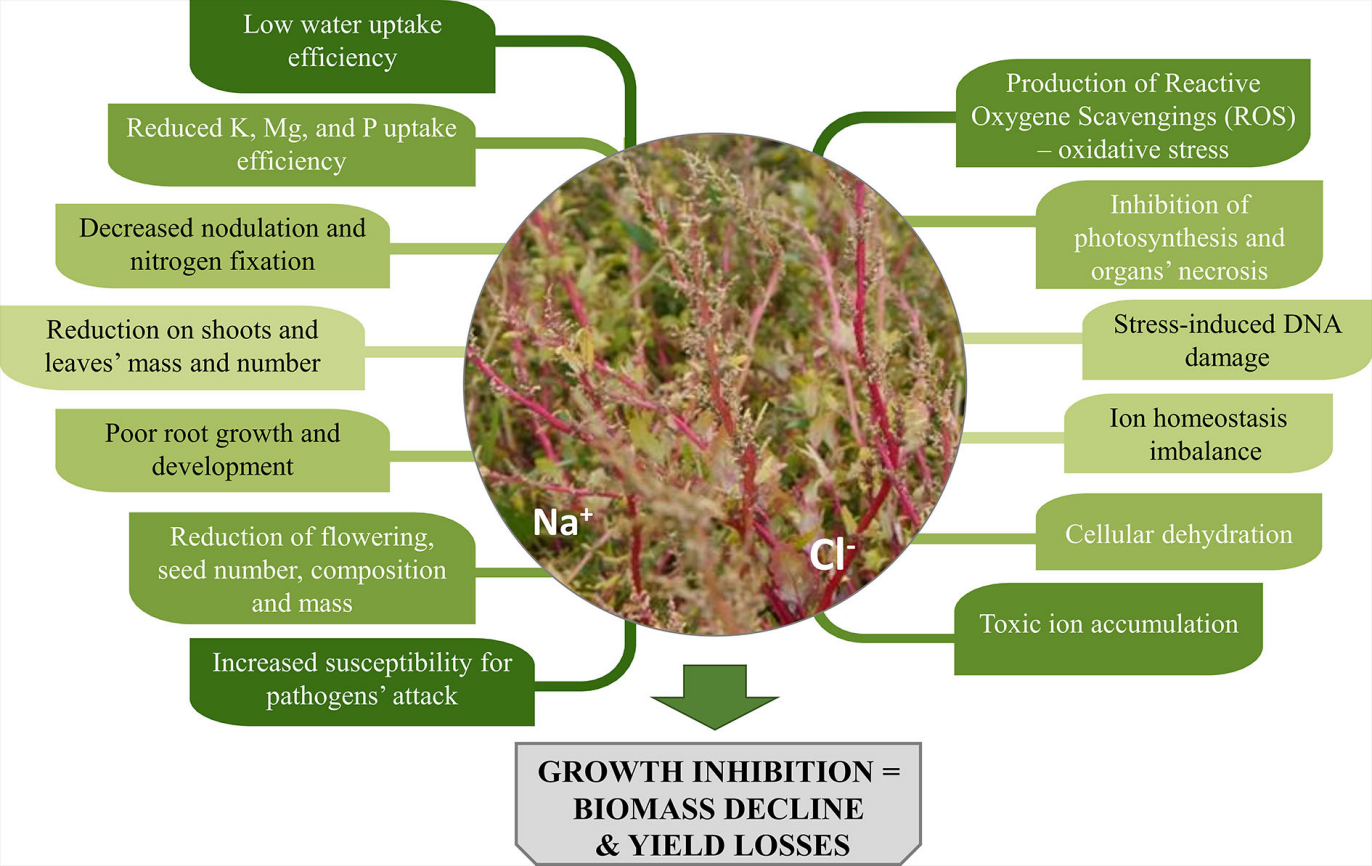
The impact of soil salinity on morphology and physiology of plants.

Salinity causes low water potential in the soil, which negatively affects plants’ water and nutrients uptake. Plants collect salts simultaneously with the water they use and often accumulate Na^+^ and Cl^-^ ions, that result toxic to plants’ cells due to ion imbalance mechanisms. What is more, enzymatic activity in cells may be disturbed. These factors trigger different responses in plants, manifested by a variety of symptoms both at cell and organ’s level (**Figure 1**).

A reduction in respiration characterises stressed plants, which also show altered assimilates distribution, inhibited photosynthesis process and lower production of new leaves. Simultaneously, increased morphological changes of organs (leaf thickening and succulence, a decrease of internode lengths), wilting, drying and even necrosis of organs and entire plants are observed (Parida and Das, 2005; Kumar and Verma, 2018). Not to mention that cultivation of crops in saline areas can adversely affect their palatability. Some plants under salinity stress might accumulate higher amounts of compounds such as oxalate and tannins, which are bitter, whereas for others, higher sugar content, and an improved flavour was observed (Masters et al., 2005; Rahneshan et al., 2018).

Different models may describe plants’ salt tolerance. One of the most popular is the Maas-Hoffman model and its modifications (van Straten et al., 2019). This mathematical tool reflects the relationship between crop yield in response to soil salinity, and for most crops, this follows a sigmoidal function. The breakpoint between the first part of the plot, being horizontal, and the second, which is sloping downward, is known as a threshold (ECt) or salt tolerance. Based on threshold and slope values, a classification in sensitive, moderately sensitive, moderately tolerant and tolerant crops can be made. There is a large variation in salt tolerance between plants’ species, from the extremely sensitive (like chickpea) to more tolerant (like cotton or beet). Among agricultural species, greens are particularly sensitive throughout the whole ontogeny of the plant, whereas the majority of cereals are highly resistant to salt impact (Munns and Rawson, 1999; Machado and Serralheiro, 2017).

In each crop, salt tolerance or sensitivity depend on the ability to uptake water and nutrients from saline soils avoiding at the same time an excessive accumulation of salt ions in the tissues. The absolute sensitivity and tolerance of plants to salinity, vary in a wide range, depending upon species, climate, soil type and its features, and agricultural practices including water management, e.g. irrigation or waterlogging methods, their frequency and intensity (Shrivastava and Kumar, 2015 and references therein).

The plants are not susceptible to salt for the whole life since their sensitivity changes during the various growth stages. In general, it was found that plants are more sensitive to salt stress when they are in their early growth stages (seedling or establishment) compared to later development phases (Machado and Serralheiro, 2017).

As mentioned, it is believed that the most sensitive to salinity stage of the plant’s growth is the germination and seedling (Läuchli and Grattan, 2007). Plants, which are better adapted to soil salinity must either have a high tolerance to salt during germination or can delay germination. Moreover, tolerance to salinity differs widely among crops without always correlating to salt-tolerances based on yield-response functions. Cotton, for instance, which is considered a salt-tolerant crop, based on lint yields, showed to be susceptible to poor stands when growing in fields irrigated with saline-sodic water (Grattan and Oster, 2003).

With maturation, plants acquire a higher tolerance to salinity. However, a prolonged exposure to salinity causes a reduction in biomass, due to lower stems numbers and smaller leaf area. A decreased leaf area commonly expresses salt-affected reduction in shoot growth, which is crucial for water uptake by the plant (Munns and Tester, 2008). In sugar beet leaves, cells’ elongation was found to be more salt-sensitive in comparison to leaf initiation related to cells’ division (Rozema et al., 2015). Hu and Schmidhalter (2007) reported that cells’ division of grass leaves was reduced by salinity. Khan et al. (2017) found in chickpea (*Cicer arietinum* L.) a reproductive failure due to reduced supply of assimilates to reproductive tissues, decreased leaf area and reduced photosynthesis, water restriction and hormonal imbalances.

Roots are the organs directly exposed to the saline environment, and they control the uptake and internal translocation of water, nutrients and salts. The anoxic situation often present in saline soils can have a more significant impact on radical architecture than salinity itself. In the case of root systems, oxygen deficiency contributes significantly to poor uptake of nutrient ions and decreased ability to toxic ions (such as Na^+^) removal (Barrett-Lennard, 2003). It is believed that roots are generally affected by excess salinity but commonly still less than aboveground organs (Rahneshan et al., 2018).

## Bacterial and Fungal Communities Associated With Roots and Rhizosphere in Saline Soils

According to Matilla et al. (2007), two selective forces of different nature are essential for microorganisms to colonise the rhizosphere: stress adaptation and the availability of particular nutrients. In saline soils, microbial communities associated with the rhizosphere, phyllosphere, and endosphere of halophytes comprehend members of Archaea and Bacteria domains and kingdom Fungi (Mukhtar et al., 2019). These communities are directly or indirectly involved in the osmoregulation of halophytes that allows them to survive under salinity stress conditions. Endophytic bacteria and fungi are those organisms whose life-cycle take place partly or entirely inside a plant. These organisms can live in the intercellular spaces of different tissues and plant’s organs (Kandel et al., 2017) without causing visible external sign of infection or a negative effect of the host (Weyens et al., 2009). Endophytes’ population in a specific environment or within a single plant may differ with tissue type, plant growth stage and dimension of the ecological niches. Several studies comparing rhizosphere and endophytic microbial communities showed how species assemblages are significantly different but also that endophytic bacteria and their communities have some peculiar, commune, traits (Kushwaha et al., 2020).

Endophytic bacterial species, for example, have larger genomes as compared to rhizosphere bacteria (Pini et al., 2011). Moreover, the diversity, richness and evenness of rhizosphere bacterial communities seem higher compared to endophytic ones (Huang, 2018). Bacterial phyla are different when considering rhizospheric soil and the plant’s tissues, with a predominance of *Proteobacteria* and *Chloroflexi* in the former and *Acidobacteria*, *Bacteroidetes*, and *Planctomycetes* in the latter (Kandel et al., 2017; Huang, 2018). However, Compant et al. (2010) pointed out that the majority of plant-associated bacteria derives from the soil environment. Some of them can penetrate plants’ roots thanks to specific mechanisms that are responsible for rhizosphere and endophytic competence. Chemotaxis, quorum sensing, flagella, antibiotic secretion, siderophore production are only some of the tools that rhizosphere bacteria need to be able to inhabit some specific plant-associated niches (Matilla et al., 2007; Compant et al., 2010). Liu et al. (2017) suggested that plant roots act as “gatekeepers” because they select soil bacteria from the rhizosphere and rhizoplane. This result in an endophytic root microbiome dominated by *Proteobacteria, Actinobacteria* and to a lower degree, by *Firmicutes* and *Bacteroidetes*, but with *Acidobacteria* and *Gemmatimonadetes* resulting almost depleted (Liu et al., 2017). The traits that characterise the bacteria that can successfully colonise and establish in endophytic niches are motility, reactive oxygen species scavenging, plant cell-wall degradation abilities. The salinity is in itself a critical environmental filter that selects species with very particular characteristics, which are probably upstream of the further selection that the rhizosphere first, and the tissues of the plant then, exert in soil microbial communities. In some studies, it has also emerged a decisive role of salinity in defining the type of endophyte associations that a plant establishes. Qin et al. (2016) provided further evidence that plants gain a higher advantage from association with a diverse microbial community (microbiome) compared with the interaction with single members of a community.

In addition to bacterial communities, also mycorrhizal symbioses showed a fundamental role in the improvement of plant nutrition, especially at the presence of environmental stresses (Qin et al., 2017). Plant-associated mycobiota belong to arbuscular mycorrhizal fungi (AMF), ectomycorrhizal fungi (EMF), non-mycorrhizal basidiomycetous fungi (NMF), and a consistent number of ascomycetous species (Zuccaro et al., 2014). Different groups, mostly based on host colonisation pattern and type of transmission, have been delimited in plant-fungal endophytes. Some fungal endophytes exhibit a vertical transmission through the host seeds or a horizontal transfer with soil- or air-borne spores (Rodriguez et al., 2009). Moreover, habitat-adapted symbiosis has been observed in some groups of fungal endophytes, and they impart a host-specific tolerance to stress in limiting environments (Qin et al., 2017). Certain groups of soil fungi have only recently been associated with the rhizosphere and endophytic situations, particularly in extreme environments or where one or more forms of stress exist (Khidir et al., 2010). Like some bacterial species, that live both as saprophytes in the soil and as endophytes, many fungal species can be isolated from both the free soil, far from the roots of plants and in close association with them (Maciá-Vicente et al., 2012). However, it must be said that several studies, conducted at different functional scales, have shown that in saline soils, the gradient of salinity as well as the presence of a high spatial heterogeneity favor the presence of species with very different functional traits and an extremely uneven distribution of communities (Maciá-Vicente et al., 2012). A similar phenomenon has been observed in the distribution of communities of bacteria in Mediterranean saline soil. Canfora et al. (2014) showed apparent differences in bacterial community distribution, diversity and composition, according to an increasing degree of soil salinity, as a consequence of a multi-scale spatial variability. A patchy distribution of vegetation structure and soil chemical properties coincided with a heterogeneous distribution of many bacterial groups. Coversely, some bacterial phyla resulted spread in the whole study area, along with the occurrence of a significant number of “salinity unrelated” phyla (*Nitrospira, Spirochaetes*). Canfora et al. (2014) hypothesized that a patchy saline environment could be “compared to a set of islands that allow the formation of different communities, separated from each other by the discontinuity of the limiting and stress factors”. Therefore, a patchy saline environment would contain not a homogeneous microbial community developed to tolerate an extreme environment, but a whole set of different communities. By comparing this evidence to the rhizosphere, it is also possible to imagine that the roots and tissues of halophilic or halotolerant plants in a saline soil represent islands of biodiversity and constitute a complicated system in which processes, at the microscale level, are particularly relevant. The living conditions within the tissues of a plant in an extreme environment such as a saline soil can reasonably be less limiting than those an organism can experience when living free in the soil. Therefore, it can be hypothesized that in saline soils, it is possible to find species as endophytes that in other systems are more easily found free in the soil. Kearl et al. (2019) isolated bacteria from halophytes (*Salicornia* and *Allenrolfea*) and observed that there were different populations in samples collected at different times of the year, with a majority of the genera, however, present independent of when the samples were collected.

Thiem et al. (2018) analysed the community structure of plant-associated endophytes of *Alnus glutinosa*, that is a dual mycorrhizal tree that forms ectomycorrhizal (EM) and arbuscular (AM) root structures, and can typically associate also with nitrogen-fixing actinomycetes. The authors sampled the plant’s root microbiome present at two forest test sites (saline and non-saline). They found that the dominant type of root microsymbionts of alder were ectomycorrhizal fungi, whose distribution depended on the site (salinity). In contrast, representatives of fungal saprotrophs or endophytes displayed the opposite tendency.

Same applies to fungi, as the Pleosporalean taxa (i.e. Pleosporales order, within the class Dothideomycetes, Ascomycota) and other generalist endophytes and epiphytes that seem particularly present in high salinity environments (Quin et al., 2017). Pleosporalean fungi like those belonging to the genera *Pleospora*, *Alternaria*, and *Phoma*, are very frequent colonisers in halophytes (Quin et al., 2017 and references therein), and in plants from arid environments (Khidir et al., 2010), and have common traits of endophytes from other adverse environments. More recent studies based on a comprehensive molecular analysis involving both fungal and bacterial communities have also highlighted a close relationship between the two. Furtado et al. (2019) examined the microbiome of the non-mycorrhizal halophyte *Salicornia europaea* and showed a significant influence of the *Salicornia* bacterial community on the fungal one, but not the other way around. They also found that the sampling season was not influencing the biodiversity. Seasonality did not appear to be an essential factor in shaping endophytic microbial communities in saline soils also from other studies (Thiem et al., 2018).

Recent studies illustrated some main mechanisms that emerged as capable of supporting directly or indirectly plant growth under saline stress (Kushwaha et al., 2020). Plants’ strategy to survive under salinity conditions comprises the synthesis and accumulation of osmolytes, as free amino acids (i.e. proline) and sugars to sustain an adequate osmotic cellular pressure needed for the functioning of cellular metabolism (Kushwaha et al., 2020). Endophytes in plants living in saline soils proved to help them accumulating osmolytes and antioxidant compounds (Vaishnav et al., 2019).

## Strategies for Increasing Salt Stress Tolerance

**Figure 2:**
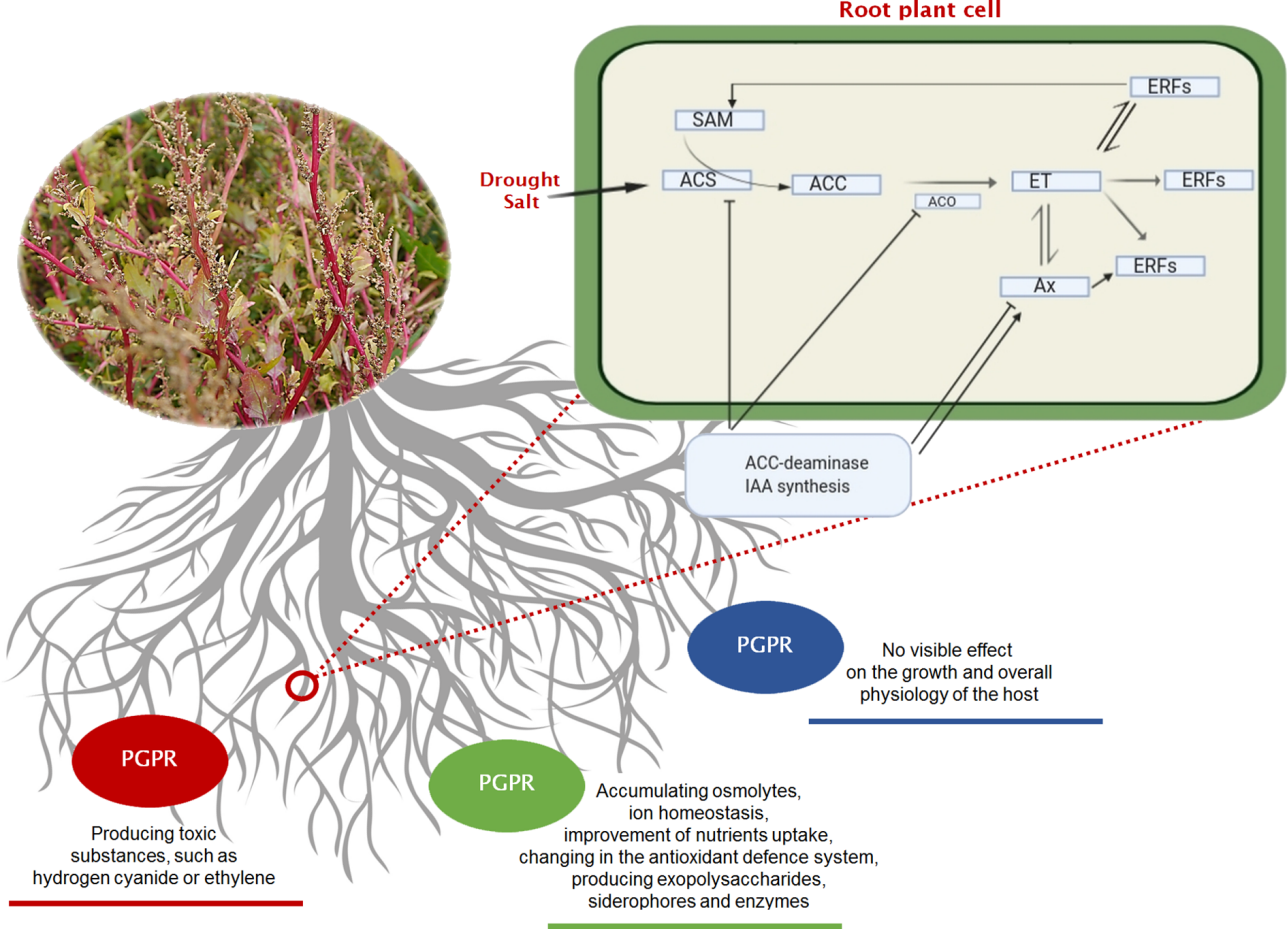
Three basic strategies of interaction (neutral—blue line, negative—red line, positive—green line) existing between the rhizobacteria and growing plants. In the negative interaction, it is highlighted the pathway of ethylene (ET) that occurs under soil stress, and the effect of mitigation that can be played by PGPRs on a plant root’s cell. Regulation of ethylene signalling and plant stress response. Ethylene pathway in plants. ACC (1-aminocyclopropane-1-carboxylic acid) the amino acid methionine is converted to SAM (S-adenosylmethionine) by the action of ACC synthase enzyme (ACS). ACC is then converted to ethylene by the enzyme ACC oxidase (ACO), triggering different ethylene response factors (ERFs). Plant growth-promoting bacteria can alter all steps of ethylene signalling. Some bacteria species can increase the ethylene levels by producing ACC oxidase (microbial ethylene-forming enzyme), by inducing ACC synthase in plant or by affecting other plant hormones indirectly. They can also modulate ethylene response by producing plant hormones that interact with ethylene signalling. Other microorganisms can also decrease ethylene production by cleaving its precursor ACC. ACC, 1-aminocyclopropane-1-carboxylate; ACS, ACC synthase; ACO, ACC oxidase; Ax, Auxine; ET, Ethylene; ERFs, Ethylene Response Factors; SAM, S-adenosyl methionine.

Halophytes are considered model plants, enabling the study of adaptive mechanisms like the induction of enzymes with antioxidant functions, the accumulation of toxic ions in their vacuoles, the storage of compatible soluble substances, occurring in the cell in response to cellular stress. Consequently, the salt-resistance genes involved in the above processes can be expressed in conventional crops, increasing their resistance to environmental salinity. So far, however, this strategy has proved to be inefficient and was implemented mainly under laboratory conditions. An equally costly and environmentally unfriendly approach in the production of salinity resistant plants is the pre-treatment of biological materials with specific, selective, chemicals, e.g. ascorbic acid, nitric oxide, H_2_O_2_, Ca^2+^, K^+^, paraquat and glutamate, silicon, phosphorus and humic acid, glycine betaine, jasmonates and salicylic acid, 5-aminolevulinic acid (El-Esawi et al., 2018) or with physical effectors like UV-B irradiation (Dhanya Thomas et al., 2020). However, these methods are not recommended for sustainable agriculture. Instead, the use of soil bacterial and fungal community colonizing plant’s roots and stimulating plant’s growth under stress conditions are promising for the increase of agricultural productivity in saline areas. Kloepper and Schroth (1978) introduced the term “rhizobacteria” to describe this microbial community collectively. Three years later, the same authors expanded the term to “plant growth-promoting rhizobacteria” (PGPR). PGPRs can be distinguished between “extracellular plant growth-promoting rhizobacteria (ePGPR)” and “intracellular plant growth-promoting rhizobacteria (iPGPR)” (Martinez-Viveros et al., 2010). The ePGPRs are bacteria that can have their niche in the rhizosphere, on the rhizoplane, or in the interstitial microenvironments of the root cortex. The iPGPRs live inside specialised nodular structures of root cells (Bhattacharyya and Jha, 2012). Some ePGPRs and iPGPRs show tolerance to a high concentration of salts and thus can also grow in saline soils and be associated to halophytes, or more in general, in niches characterized by low water potentials due to salt stress or to a dry climate (Khan et al., 2019) (**Supplementary Material Table 1**). Salt-tolerant PGPRs (ST-PGPR) include mainly bacteria of the genus *Bacillus, Pseudomonas, Enterobacter, Agrobacterium, Streptomyces, Klebsiella* and *Ochrobactrum* (Sharma et al., 2016). Whipps (2001) roughly distinguished between three strategies of interaction of the rhizobacteria with the plants: neutral, negative, or positive. Neutral interaction means that the rhizobacteria, although living in the plant’s rhizosphere have no visible effect on the growth and metabolism of the host (Beattie, 2006). Positive interactions comprise those ST-PGPRs (salt-tolerant plant growth promoting rhizobacteria) that have a promoting effect on plant physiology and tolerance to salinity (**Figure 2**). The ST-PGPRs, in turn, from a utilitarian point of view, can be subdivided into biofertilizer microorganisms, phytostimulators, and biopesticides. Negative interactions comprehend all those situations where the bacteria are phytopathogenic or produce substances toxic to plants (i.e. hydrogen cyanide or ethylene). However, sometimes, the secretion of the toxic substance, such as hydrogen cyanide by PGPRs, is also beneficial as it is one of the many ways to protect the plant from phytopathogens. ST-PGPR can improve plant’s growth and tolerance to salinity stress through the accumulation of osmolytes (proline, trehalose, and glycine betaines) (**Supplementary Material Table 2**), the production of phytohormones (auxins, gibberellins, cytokinins), ion homeostasis, the improvement of nutrients uptake (N_2_ fixation, solubilising of P, K, Zn, and Si), the activation of plant’s antioxidative enzymes, and the synthesis of ACC deaminase, indole-3-acetic acid (IAA), exopolysaccharides, and siderophores (Egamberdieva et al., 2019).

Plant growth-promoting fungi (PGPFs) are soil-borne, non-pathogenic, saprophytic microorganisms. This group contains several fungal taxa, and among the most quoted genera, especially in saline environments, there are *Penicillium* spp., *Fusarium* spp., *Alternaria* spp., *Aspergillus* spp., *Sclerotium* spp., and *Phoma* spp., which colonise plants roots and form symbiotic interactions with them (Gangwar et al., 2017; Naziya et al., 2020). Pereira et al. (2019), for example, surveyed the culturable endophytic mycobiota of *Festuca rubra*, a perennial grass diffused in coastal environments with low nutrient availability, wind, and salinity. Taxa belonging to *Fusarium*, *Diaporthe*, Helotiales, *Drechslera*, *Slopeiomyces*, and *Penicillium* were constant inhabitants of the plant’s roots (occurred in more than 20% of the plants the authors analysed). Seventy-one point eight percent of the strains they could culture were halotolerant. When the authors used the isolates to inoculate the grass *Lolium perenne*, a *Diaporthe* strain increased leaf biomass production under both normal and saline watering regimes (200 mM NaCl). PGPFs enhance the plant growth directly and indirectly by the production of IAA, siderophore, cellulase, chitinase, gibberellins, and increasing phosphorus solubilisation and availability (Mishra et al., 2017; Naziya et al., 2020).

High salinity increases susceptibility to various phytopathogens and promotes some fungal soil-borne diseases in plants. Crops protection against pathogens is extremely difficult, because the use of chemicals agents e.g. fungicides, bactericides and nematicides has a negative impact on the environment and organisms while increases the costs of cultivation (Egamberdieva et al., 2017; Sharma et al., 2017). An alternative to the use of chemicals is the use of PGPRs and PGPFs, which could synthesise the aforementioned lytic enzymes, siderophores, IAA, and antibiotics. These compounds could reduce and inhibit phytopathogens that cause plant infections (Labuschagne et al., 2010; Murali et al., 2012; Gangwar et al., 2017). *Trichoderma* isolates widely used in bio-fungicidal formulations can be ineffective at high salinity conditions, since most of these fungal species have a low osmotolerance (Mohamed and Haggag, 2006; Bheemaraya et al., 2013). However, some studies targeted halotolerant biological control fungi. This is the case of the experiments by Gal-Hemed et al. (2011) who isolated *T. atroviride* and *T. asperelloides* from the Mediterranean sponge *Psammocinia* sp. and used them to reduce *Rhizoctonia solani* disease in beans, and more recently the study by Sánchez-Montesinos et al. (2019). They isolated *Trichoderma* strains from the seagrass *Posidonia oceanica* and evaluated its capacity to control the disease caused by *Pythium ultimum* in melon seedlings under various levels of salt stress.

### Osmoprotectants

In response to salinity stress, ST-PGPRs accumulate many metabolites called compatible (organic) solutes, among which amino acids and derivatives (e.g., glutamate, proline, peptides, and N-acetylated amino acids), quaternary amines (e.g., glycine betaine and carnitine), sugars (e.g., sucrose and trehalose), and tetrahydro pyrimidines (ectoines). These metabolites enhance the stability of protein conformation, the balance of cell redox condition, cytosolic pH, complex II electron transport, membrane integrity, and the activity of enzymes such as ribulose bisphosphate carboxylase/oxygenase (RUBISCO) (Saghafi et al., 2019). Moreover, the storage of osmolytes represents a successful stress response mechanism that helps the bacteria to limit water loss, increasing their cytoplasmic concentration of K^+^. In some halophilic bacteria, the internal concentration of osmoprotectants due to salt stress may reach up to 1 M (Saum and Müller, 2007). Rodriguez-Salazar et al. (2009) observed that *Azospirillum* spp. accumulates proline, trehalose, and glycine betaine as a mechanism for protection against osmotic stress. Kushwaha et al. (2019) found in *Halomonas* sp. that accumulation of betaine suppresses the *de novo* synthesis of ectoine at low NaCl concentrations, however, at higher NaCl concentrations the amount of ectoine is significantly larger than betaine. It means that the salinity stress transcriptionally up-regulates ectoine accumulation. The expression of the genes *pro*H, *pro*J, and *pro*A involved in proline biosynthesis was induced in some of the halophilic bacteria at higher salt concentration, leading to the highest accumulation of proline (Saum and Müller, 2007). Also, several reports determined the link between proline accumulation and pyrroline-5-carboxylate synthase (P5CS) gene expression level after PGPR inoculation and hypothesized that bacterial treatment upregulates the P5CS gene expression in plant roots, causing intracellular storage of free proline (Kim et al., 2007; Kumari et al., 2015a; Kumari et al., 2015b). Many ST-PGPRs showed a high expression of genes implicated in trehalose biosynthetic pathways (e.g. trehalose 6-phosphate gene) (Qin et al., 2018). Trehalose is an osmoprotectant, and its role in salt-stress tolerance has been well documented (Garg et al., 2019; Orozco-Mosqueda et al., 2019; Shim et al., 2019). Figueiredo et al. (2008) reported that *Rhizobium tropici* and *Paenibacillus polymyxa* were modiﬁed to overexpress trehalose 6-phosphate gene and were co-inoculated in *Phaseolus vulgaris* plants resulting beneficial for plants grown under saline stress, with higher nodulation and N content. A differential gene expression analysis of the tissues of the nodules compared to normal roots revealed upregulation of stress tolerance genes, which suggested that extracellular trehalose works as an osmoprotectant, including tolerance to salinity (Figueiredo et al., 2008).

### Ion Homeostasis

One strategy used by bacteria to limit salt uptake, also by plants, is by trapping cations in their exopolysaccharide matrix. This mechanism results in an altered root structure with the formation of extensive rhizo-sheaths (agglutinated soil that adheres to roots when they are removed from the pot or field). Moreover, at the rhizosphere level, it has been found differential regulation of the expression of genes involved in ion aﬃnity transporters. PGPR often impact the mineral nutrient exchange of both macro and micronutrients as a strategy to react to nutrient imbalance due to a higher uptake of Na^+^ and Cl^−^ ions (Saghafi et al., 2019). Both fungi and bacteria can help plants to keep cellular ion homeostasis and sustainable K^+^/Na^+^ ratios in shoots. This has been documented as a mechanism that reduces Na^+^ and Cl^−^ accumulation in leaves, by increasing Na^+^ exclusion *via* roots and boosting the activity of high-affinity K^+^ transporters (Ilangumaran and Smith, 2017). Zhang et al. (2008a) reported that the inoculation of *Arabidopsis thaliana* with *B. subtilis* moderated the adverse effects of salinity by regulating HKT1 potassium transporter. This bacterium also stimulated the overexpression of the AtHKT1 gene, expressing for a high-compatibility transporter for potassium ion, in *Arabidopsis* under conditions of salt stress. *Puccinellia tenuiﬂora*, a salt-excluding halophytic grass, when inoculated with *B. subtilis* showed lower levels of Na^+^ accumulation. The plant at the same time upregulated plasma membrane Na+/H+ transporters SOS1, and HKT-type protein and tonoplast Na+/H+ antiporters genes. However, one of the HKT genes was downregulated in roots under high salt concentrations (Zhang et al., 2008a; Niu et al., 2016). This showed how the bacterium synergistically regulated Na+ homeostasis by controlling Na+ transport systems at the whole-plant level under both lower and higher salt conditions, differentiating the mechanisms at play in case of high or mild salinity conditions. According to Rojas-Tapias et al. (2012), the inoculation of auxin-producing strains of *Azotobacter* in maize plants exposed to saline stress resulted in better K^+^ uptake and Na^+^ exclusion from plant’s tissues.

Moreover, the authors showed that after PGPR inoculation chlorophyll, proline, and polyphenol contents in maize leaves increased along with a better general plant stress response (Rojas-Tapias et al., 2012). Ilangumaran and Smith (2017) reported that in many studies on the interaction between plants and PGPR species, the genes involved in ion homeostasis showed differential expression under saline stress. For example, in an experiment where *Arabidopsis thaliana* was treated with *Burkholderia phytoﬁrmans* the expression of both bacterial and plant’s genes involved in ion homeostasis (KT1, HKT1, NHX2, and SOS1) was rapidly altered as a result of an imposed saline stress (Pinedo et al., 2015).

Also, fungi can play a decisive role in a plant’s ability to improve nutrients’ uptake and regulate the osmotic balance in soils affected by salinity. Arbuscular mycorrhizal fungi are plants symbionts that increase root phosphorus uptake and confer the mycorrhizal plants’ tolerance to salinity. Romero-Munar et al. (2019) demonstrated that in *Arundo donax* a commercial inoculum containing the arbuscular mycorrhizal *Rhizophagus* *intraradices* and *Funneliformis mosseae* improved the nutritional status by enhancing nutrient use efficiency. The authors suggested that increased use efficiency of phosphorus could have improved ion (Na^+^ and K^+^) uptake and allocation. Arbuscular mycorrhizal fungi have also been reported to enhance the ability of wheat plants to modulate the reactive oxygen scavenging system when coping with salinity stress (Talaat and Shawky, 2011) (**Table 1**).

**Table 1:** The effect of halophilic PGPRs and PGPFs on alleviating salt stress in halophytes.

Host halophyte	Microorganisms	Bacterial/Fungal activity	Plant response	References
*Arthrocnemum macrostachyum* L. (glaucous glasswort)	*Bacillus alcalophilus*, *Bacillus thuringiensis*, *Gracibacillus saliphilus*	IAA production, siderophore, and phosphate solubilisation	Mitigating the effects of high salinity on plant growth and physiological performance	Navarro-Torre et al., 2017
*Aster tripolium* L. (sea aster)	*Bacillus cereus*, *Serratia marcescens*	IAA production, siderophore production, N_2_ fixation, and ACC deaminase activity	–	Szymańska et al., 2016
*Atriplex leucoclada* L. (orache)	*Arthrobacter pascens*	Phosphate solubilization and siderophore production	Increase in root and shoot length, fresh and dry weight, accumulation of osmolytes (e.g., sugar, proline), increase in activity of antioxidant enzymes	Ullah and Bano, 2015
*Bassia indica* L. (Kochia indica)	*Bacillus subtilis*	IAA and ACC deaminase production	Improving root and shoot growth, total lipid content, the phospholipid fraction, photosynthetic pigments (chlorophyll a and b and carotenoid contents); 200 mM NaCl	Abeer et al., 2015
*Beta vulgaris* L. (beet)	*Micrococcus yunnanensis*, *Planococcus rifietoensis, Variovorax paradoxus*	ACC deaminase production	Improving germination and plant biomass, higher photosynthetic capacity and lower stress-induced ethylene production; 50–125 mM NaCl	Zhou et al., 2017
*Brassica napus* L. (canola)	*Rhizobium legominozaroum*, *Sinorhizobium mellilote*,	IAA, ACC deaminase production and phosphate solubilizing	Increasing in all of the growth indices (plant height, root and shoot dry weight), nutrient uptake and restricted availability for plants	Saghafi et al., 2018
	*Bacillus aryabhattai, Brevibacterium epidermidis*, *Micrococcus yunnanensis*	IAA, ACC deaminase, ammonia production, nitrogen fixation, phosphorus and zinc solubilization, thiosulfate oxidation, production of extracellular hydrolytic enzymes	40% increase in root elongation and plant dry weight; 150 mM NaCl	Siddikee et al., 2010
	*Enterobacter cloacae, Paenibacillus xylanexedens*	IAA, ACC deaminase production	Enhance plant root elongation	Yaish et al., 2015
*Catharanthus roseus* L. (Madagascar periwinkle)	*Achromobacter xylosoxidans*	ACC deaminase production, nitrogen fixation, increasing the level of antioxidative enzyme	Decreasing stress ethylene level; influence on germination, plant height and root weight	Karthikeyan et al., 2012
*Cicer arietinum* (chickpea)	*Halomonas variabilis*, *Planococcus rifietoensis*	EPS production	Increasing the plant growth and soil aggregation	Qurashi and Sabr, 2012
*Coriandrum sativum* (coriander)	*Pseudomonas pseudoalcaligenes*, *Pseudomonas putida*	P-solubilization, photosynthetic pigments, IAA, ACC deaminase production and increasing the level of POD	Improving plant growth and root system	Al-Garni et al., 2019
*Glycine max* L. (soybean)	*Pseudomonas* sp.	EPS production	Effects on the elongation of shoots and roots, number of lateral roots, shoot and root fresh weight, and decreased Na+/K+ ratio under salinity stress.	Kasotia et al., 2016
*Helianthus annus* L. (sunflowers plant)	Bacterial strains	ACC deaminase production	Increasing plant height, shoot dry weight and root dry weight, phosphorus, potassium contents, and K+/Na+ ratio in the shoot	Kiani et al., 2015
*Prosopis strombulifera* (creeping screwbean)	*Achromobacter xylosoxidans*, *Bacillus licheniformis*, *Bacillus pumilus*, *Brevibacterium halotolerans*, *Lysinibacillus fusiformis*, *Pseudomonas putida*	IAA production, siderophore production, N_2_ fixation, ACC deaminase activity, gibberelin production, protease and antifungal activity	–	Sgroy et al., 2009
*Salicornia brachiata* (glasswort)	*Brachybacterium saurashtrense*	IAA production, siderophore production, N_2_ fixation, ACC deaminase activity	–	Gontia et al., 2011
	*Agrobacterium tumefaciens*, *Brachybacterium* *saurashtrense*, *Brevibacterium* *casei*, *Haererohalobacter* sp., *Zhinguelliuella* sp.	IAA production, phosphate solubilization, siderophore production, N_2_ fixation, ACC deaminase activity	Increase in amino acids, IAA, content of Ca^2+^, P, N of the inoculated plants; in the percentage of water content in roots and shoots in inoculated plants; increase total biomass; increased in plant length and dry weight compared to un-inoculated plants	Shukla et al., 2012
Solanum lycopersicum L. (tomato)	*Leclercia adecarboxylata*	IAA, ACC deaminase and osmoprotectants production	Improving plant growth	Kang et al., 2019
	*Achromobacter piechaudii*	ACC deaminase production	Increasing fresh and dry weights of tomato seedlings grown in the presence of up to 172 mM NaCl salt	Mayak et al., 2004
*Suaeda salsa* L. (seepweed)	*Pantoea agglomerans*, *Pseudomonas oryzihabitans, Pseudomonas putida*	Gibberellic acid production, IAA production, ACC deaminase activity, siderophore production, abscisic acid production, and antifungal activity	–	Teng et al., 2010
*Triticum aestivum* L. (wheat)	*Bacillus methylotrophicus*, *Bacillus siamensis*, *Bacillus* sp.	IAA, ACC deaminase, and EPS production	Influence on the germination rate of wheat seedlings, root and shoot length, and photosynthetic pigments.	Amna et al., 2019
	*Klebsiella* sp. *Serratia* sp.	Auxin and siderophores production	Increasing the plant biomass; 0.25 M and 0.45 M NaCl	Acuña et al., 2019
	*Bacillus gibsonii*, *Bacillus* sp., *Halomonas* sp., *Oceanobacillus oncorhynchi*, *Zhihengliuella* spp.	IAA, ACC deaminase and ammonia production, nitrogen fixation and phosphate solubilization	Increasing root and shoot length and total fresh plant weight	Orhan, 2016
*Triticum turgidum* subsp. *durum* (durum wheat)	rhizospheric and endophytic bacteria	Nitrogen fixation, ACC deaminase and auxin production, inorganic phosphate solubilization and siderophore production	Improving survival in inoculated plants under high salinity stress conditions, faster germination rates, and seedling growth	Albdaiwi et al., 2019
*Zea mays* L. (maize)	*Serratia liquefaciens*	antioxidant enzymes (APX, CAT, SOD, POD), non-enzymatic redox antioxidants (ascorbic acid and glutathione) induction and osmoprotectants production	Reduction of oxidative stress markers and increase the maize growth and biomass production along with better leaf gas exchange, osmoregulation, antioxidant defence systems, and nutrient uptake under salt stress (80 and 160 mM NaCl)	El-Esawi et al., 2018
	*Pantoea alli*, *Pseudomonas reactans*, *Rhizoglomus irregulare*	Osmotic adjustment	Consortium tended to mitigate ion imbalances in plants across the gradient of NaCl (0–5 g/kg of soil), promoting maize growth and nutritional status.	Moreira et al., 2020
*Arundo donax* L. (giant reed)	*Rhizophagus intraradices* *Funneliformis mosseae*	Improving the nutritional status of plants by enhancing nutrient use efficiency	Improving plant growth (1, 75, and 150 mM NaCl)	Romero-Munar et al., 2019
*Brassica napus* L. (canola)	*Trichoderma parareesei*	Increasing the expression of genes related to the pathways of ethylene	Increasing rape seed yield	Poveda, 2020
*Lolium perenne* (ryegrass)	*Diaporthe* strain S69	Ion homeostasis	Promoting leaf biomass production (200 mM NaCl)	Pereira et al., 20192019
	*Arthrinium gamsii* sp, *Stereum gausapatum* sp., isolated from *Salicornia europaea*	Siderophores, polyamines, IAA and cellulolytic, proteolytic, lipolytic and chitinolytic enzymes production	Increasing the length, fresh and dry weights of the shoots and roots	Furtado et al., 2019b
*Sesamum indicum* L. (sesame)	*Penicillium* sp.	Chlorophylls, proteins, amino acids, and lignans production	Increasing the length of shoot and root, and fresh and dry seedling weight (150 mM NaCl)	Radhakrishnan et al., 2014
Wheat (cv. Yongliang 4)	*Trichoderma longibrachiatum*	Improvement of the antioxidative defense system and gene expression in the stressed plants	Increasing root and shoot length and total fresh plant weight	Zhang et al., 2016
Wheat	*Trichoderma reesei*	Flavonoid, phenolic compounds, phytoharmones, including IAA and gibberellic acid production	Improving plant growth. Increased amount of chlorophyll a and b, carotenoids	Ikram et al., 2019

Symbiotic fungi can modulate gene expression of the host plant to modify its phenotype in order to improve the tolerance to abiotic stress factors caused by soil salinity (Khan et al., 2013). Molina-Montenegro et al. (2020) showed that inoculation of plants’ roots with Antarctic fungal endophytes improves growth and survival by changing the expression of a gene responsible for Na^+^/H^+^ antiporters proteins integrated with vacuolar membranes. In particular, the NHX proteins are involved in the maintenance of cell turgor through ionic balance control and are associated with the capacity of accumulating Na^+^ inside vacuoles.

### The Improvement of Phosphorus, Potassium, and Zinc Solubilisation

Phosphorus (P) is one of the essential macronutrients for plants, although its availability is limited due to its low solubility. P is required as an essential nutritional element for photosynthesis, energy transfer, biosynthesis of macromolecules and respiration (Fernandez et al., 2007). The average content of phosphorus ions in the soil is 0.05% (w/w). Still, often only 0.1% of the total P is available to plants because of its precipitation in soil (Etesami and Beattie, 2018). Salinity leads to depletion and sedimentation of absorbable phosphorus. Phosphate-solubilising halotolerant PGPRs (*Bacillus, Pseudomonas*, *Achromobacter, Alcaligenes, Brevibacterium, Serratia, Xanthomonas*, and *Rhizobium*) provide an opportunity to enhance P availability to plants without deteriorating soil salinity levels. These microorganisms can hydrolyse inaccessible phosphorus forms into absorbable forms *via* various mechanisms like chelation, ion exchange, and acidification by secreting low molecular weight organic acids, such as gluconic acid, citric acid, succinic acid, propionic acid, and lactic acid (Choudhary, 2012; Etesami and Beattie, 2018; Saghafi et al., 2018). In salt-affected soils, the inoculation of wheat with *Bacillus aquimaris* increased plant P content under salinity stress (Upadhyay and Singh, 2015). Khan et al. (2019) identified highly salt stress-tolerant strains of *Arthrobacter woluwensis*, *Microbacterium oxydans*, *Arthrobacter aurescens*, *Bacillus megaterium*, and *Bacillus aryabhattai*, which showed to increase phosphate uptake in several plants: *Artemisia princeps, Chenopodium ficifolium, Echinochloa crus-galli*, and *Oenothera biennis* (Khan et al., 2019). Salcedo et al. (2014) identiﬁed seven best phosphate-solubilising actinobacteria strains out of the 57 strains that they isolated from soil. El-Tarabily and Youssef (2010) screened the mangrove *A. marina* rhizosphere and identiﬁed 129 bacterial strains capable of solubilising rock phosphate. In particular, *Oceanobacillus picturae* showed to be able to mobilize 97% of the available mineral P. Many other genera of bacteria isolated from halophytes (i.e. *Arthrobacter, Bacillus, Azospirillum, Vibrio, Phyllobacterium*) were found capable to implement P absorption in halophytes under salinity stress (Banerjee et al., 2010; Yasmin and Bano, 2011). Bashan et al. (2000) showed that in the leaves of halophytes inoculated with halotolerant PGPRs, as species of the genera *Azospirillum*, *Vibrio, Bacillus*, and *Phyllobacteriu*m, the P content increased. Vaishnav et al. (2016) used a hydroponic system to demonstrate how insoluble phosphate-solubilising bacteria that solubilise sedimentary phosphorous actively increased the availability of assimilable P to plants in salinity stress conditions. Yadav et al. (2011) reported the contribution of *Aspergillus niger*, *Penicillium citrinum*, and *Trichoderma harzianum* in phosphate solubilisation and their beneficial effects on chickpea growth. Amongst studied fungal genera, the highest P-solubilizing ability was attributed to *Aspergillus* and *Trichoderma* species. Ceci et al. (2018) showed that many saprotrophic fungi could mobilize P from insoluble forms according to a variety of mechanisms, with strains of *Rhizopus stolonifer* var. *stolonifer*, *Aspergillus niger* and *Alternaria alternata* among the best performing strains in terms of amounts of insoluble phosphate solubilisation.

Apart from phosphorus, another essential nutrient ingredient for plants is potassium (K). This element plays a vital role in plant metabolism and improves the quality of the crop production due to its role in grain filling, and in promoting disease resistance, leading to a higher resistance of plants to stress. The concentration of potassium in the soil solution is usually 1%–2% (Sindhu et al., 2010). The potential of soil application of potassium solubilising microorganisms (KSBs) is widely studied, especially in saline soils where this element is even less available to plants. These bacteria solubilise potassium-containing minerals (mica and orthoclase) by producing tartaric, succinic, citric, oxalic, and alpha-ketogluconic acids (Saghafi et al., 2019). Singh et al. (2010) reported that *Bacillus mucilaginosa*, *Azotobacter chroococcum*, and *Rhizobium* sp. were able to increase potassium absorption by wheat and corn.

Several authors showed that also fungi, especially ectomycorrhizal species, can weather silicate minerals to extract nutrients like P actively, K, Ca, Mg, and Fe, in particular under conditions of nutrient limitation. Mycorrhizal (ecto- and endo-) contribution to K+ acquisition by plants has also been demonstrated (Benito and Gonzalez-Guerrero, 2014). Fungi are already used at industrial level to mobilize or precipitate also other metals, like Cu, Mn, Zn, even though they are poorly applied for plant nutrition. There are studies, however, for the application of fungi and bacteria as solubilizers of specific nutrients, and some bacteria are already applied in fertilization treatments to this aim.

Zinc deficiency, for example, is a significant problem for the plant, especially in saline arid and semi-arid soils. Plants absorb Zn mainly in the form of Zn^2+^, zinc hydrate, and organic zeolite and use it in biochemical reactions, for the stability of biological membranes, the activity of oxidative and carbonic anhydrase enzymes and the synthesis of the enzyme auxin (Broadley et al., 2007; Alaghemand et al., 2018). The most important method to provide plants with Zn is the application of rhizobacteria together with Zn-containing fertilizers. These bacteria can increase the solubility of poorly soluble Zn compounds by employing different mechanisms, such as chelation by siderophore (Tariq et al., 2007), reduction of soil pH by the production of organic acids (2-ketogluconic acid, gluconic acid) and proton secretion (Subramanian et al., 2009). Abaid-Ullah et al. (2015) reported that *Serratia* sp. could increase wheat yield through solubilisation of ZnO under different climates.

### Biological Nitrogen Fixation by PGPRs

Halophytic crop species used in agriculture can be limited by the lack of available nitrogen often affecting saline soils. PGPRs can fix nitrogen through symbiotic and non-symbiotic mechanisms (Saghafi et al., 2019). The first method involves the formation of nodes in the host roots by bacteria which results in nitrogen content of approximately 65% of the total nitrogen assimilation by plants (Rajwar et al., 2013). The other group of nitrogen-fixing bacteria, including *Azospirillum*, *Azotobacter, Burkholderia, Herbaspirillum, Bacillus*, and *Paenibacillus* is not plant-specific (Goswami et al., 2015). Salt-tolerant N_2_-fixing PGPRs are an essential source of available N in saline soils, and the amount of nitrogen fixed by these bacteria has been estimated as 20-30 kg h^-1^ year^-1^ (Oberson et al., 2013). The potential benefits of nitrogen-fixing strains to halophytes and salt-sensitive crops underline the need of implementing the studies on N_2_-fixing halotolerant PGPRs to be used as boosters in saline soil-based agriculture (Goswami et al., 2015; Ilangumaran and Smith, 2017).

### Siderophore Production

Some strains of bacteria produce siderophores, especially in the rhizosphere, this increases plant growth and prevent phytopathogens from proliferation by inhibiting them from accumulating iron (Scavino and Pedraza, 2013). Siderophores are Fe(III)-chelating compounds, usually small and with high-affinity so that plants, in need for iron nutrient, quickly access the iron-siderophore complexes. Iron is an integral part and cofactor of enzymes involved in plants’ respiration, photosynthesis, N_2_ fixation and many other biochemical processes (Abbas et al., 2015; Etesami and Beattie, 2018). Bacterial siderophores have a higher affinity for iron than fungal pathogens, which require iron for their metabolism and plants’ infecting mechanisms (Miethke and Marahiel, 2007). Many halotolerant PGPRs and PGPFs, particularly those isolated from halophytes (**Table 1**) produce iron siderophores. Among biocontrol agents, the strains belonging to *Pseudomonas* sp. secreting non-fluorescent and fluorescent siderophores such as pyochelins and pseudobactins are most effective competitors of Fe^3+^. The potential uses of siderophore producing bacterial strains have been reported in the suppression of fungal pathogens of rice and wheat (Labuschagne et al., 2010). Moreover, pyoverdine synthetised by *P.* *aeruginosa* under low-iron stress condition could inhibit the growth of *Aspergillus flavus, A. oryzae, F. oxysporum*, and *Sclerotium rolfsii* (Manwar et al., 2004).

Also, fungi, however, produce siderophores that can act as protectants against plants’ pathogens. Fungal strains belonging to *Aureobasidium* and *Emericellopsis* genera synthesise siderophores, which are part of the biocontrol strategies occurring in *Salicornia* plants (Furtado et al., 2019).

### IAA Production

Indole-3-acetic acid (IAA) is the most common plant hormone of the auxin class, and it regulates various aspects of plant growth and development. IAA acts as an effector molecule between bacteria and IAA producing plants, and in bacterial-bacterial interactions (Spaepen and Vanderleyden, 2011). It is involved in many processes such as seed germination, root system development, or increasing plant tolerance to stress conditions (Aeron et al., 2011). IAA-producing microorganisms increase the root growth and root length of plants, which contributes to a greater root surface area enabling the plant to acquire more nutrients from the soil (Boiero et al., 2007). Tryptophan is the IAA precursor in most biosynthetic pathways. However, several reports indicate the possibility of IAA synthesis in tryptophan-independent reactions (Sitbon et al., 2000; Saghafi et al., 2018).

The positive impact of IAA produced by PGPRs on the growth of various plants in conditions of salinity stress has been determined. For example, IAA-producing, halotolerant and halophilic bacteria significantly affected root and shoot elongation and freshly available mass of *Triticum aestivum* plants under salt stress conditions (Orhan, 2016). Also, *Brassica napus* L. seedlings inoculated with IAA producing *Rhizobium* bacteria showed an improved growth rate under salt stress, regarding especially plant height, as well as root and shoot dry weight (Saghafi et al., 2018). It has been confirmed that the salt-tolerant *B. subtilis* promotes the growth and fitness of Indian bassia plants (*Bassia indica*) under salt stress by providing an additional supply of IAA, and induces salt stress resistance by reducing ethylene levels. Inoculation of unstressed and salt-stressed Indian bassia with *B. subtilis* has significantly improved root and shoot growth, total lipid content, the phospholipid fraction, the content of photosynthetic pigments and also increased oleic, linoleic, and linolenic acids in plant leaves, as compared to uninoculated plants (Abeer et al., 2015). IAA-producing bacteria are also involved in suppression of plant disease-causing pathogenic fungi. *Pseudomonas extremorientalis* and *P. aureantiaca* were successfully used for control cucumber root infection caused by *F. solani* (Egamberdieva et al., 2014). Other examples are given in **Table 1**. Sodium chloride (NaCl) induces a decline in the IAA level in rice seedlings (Sakhabutdinova et al., 2003). Auxin activates the transcription of various genes known as primary auxin response genes in *Arabidopsis*, rice and soybeans (Hagen and Guilfoyle, 2002). Auxin negatively regulates the expression of the rice gene adenosine phosphate isopentenyltransferase (OsIPT) that encodes a key enzyme in CTK biosynthesis in nodes, thus inhibiting the growth of tiller buds in rice (Liu et al., 2011). Therefore, the identification of new genes that respond to high saline conditions offers investigators the opportunity to develop new approaches to select varieties with different mechanisms of tolerance to salinity stress (Zhu, 2002).

### ACC Deaminase Production

**Figure 3:**
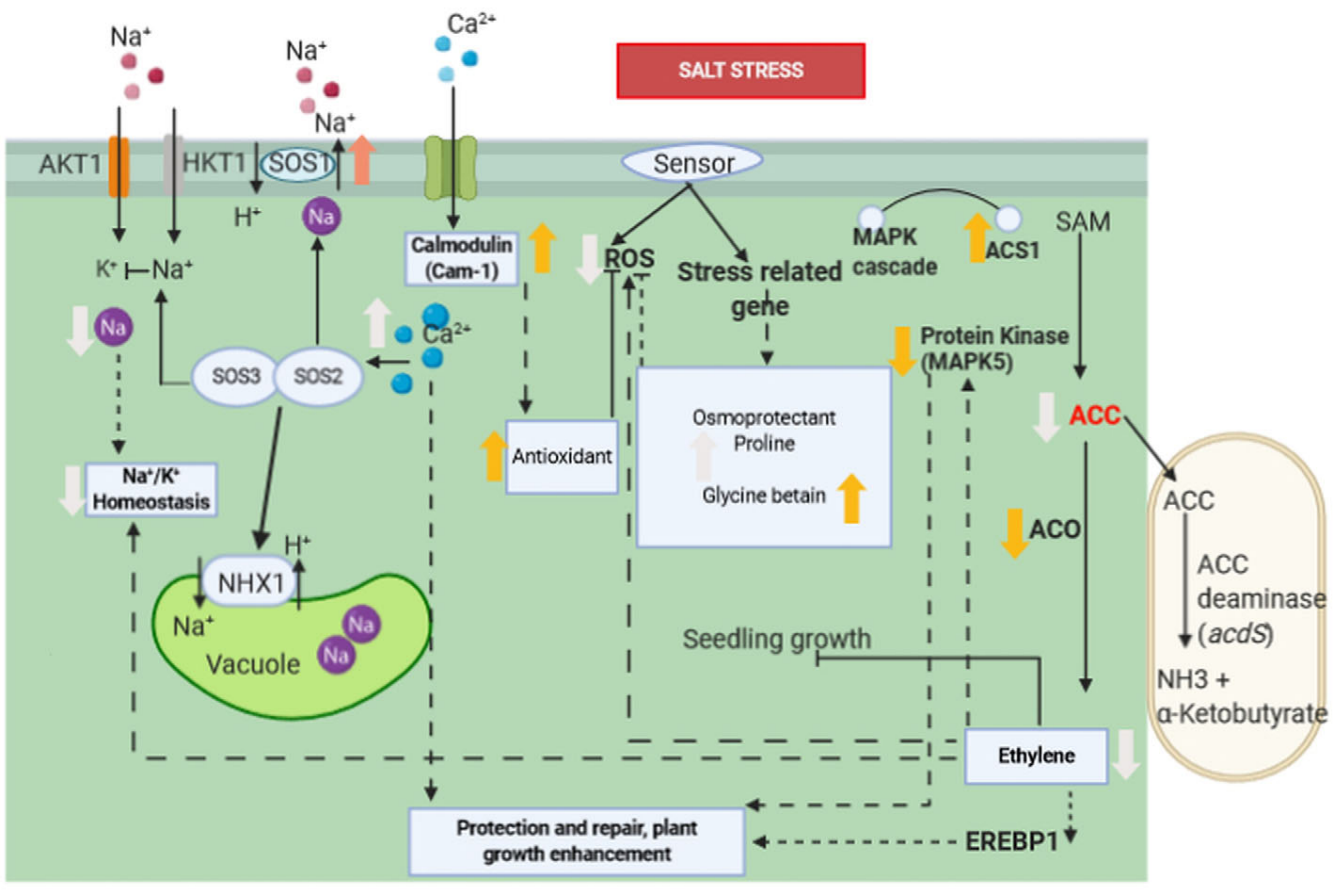
Molecular interaction of ACCD-producing endophytic bacteria associated with plant roots under saline stress. Salt stress induces the ethylene biosynthesis pathway by upregulation of ACS1. However, ACC is consumed as a result of the activation of acdS gene encoding ACCD of PGPR, whereas ACO1 and EREBP1 are down-regulated and ethylene production is reduced as a consequence. The reduction of ethylene induces a lower expression of MAPK5 and reduces the accumulation of ROS. Increase in proline, betaine, and glycine improves salt tolerance in plant’s roots. Ca^2+^content is increased, and Na^+^/K^+^ ratio is decreased, which are correlated with up-regulation of Cam1, SOS1, and NHX1 genes. Ca^2+^ signal activates the SOS3/SOS2 protein kinase complex, which negatively regulates the activity of Na^+^ ion channel. Association of Ca^2+^ and calmodulin activates antioxidant enzymes which subsequently inhibits ROS. Bold orange arrow indicates gene regulation, bold white arrow indicates plant physiological regulation, black arrow indicates positive regulation, dashed arrow indicates indirect positive regulation, black line with bar-end indicates inhibition and dashed line with bar-end indicates indirect inhibition.

The production of ACC deaminase enzyme is an essential mechanism for the direct promotion of plant growth by PGPRs. Bacterial IAA affects the level of ethylene in plants by increasing the activity of ACC deaminase, catalysing the hydrolysis of 1-amino-cyclopropane-1-carboxylic acid (ACC), an ethylene precursor, to ammonia and α-ketobutyric acid (Glick, 2005; Etesami et al., 2015). In fact, under stress conditions, including salinity, ethylene level increases in the plant and 1-aminocyclopropane-1-carboxylate (ACC) is more consistently enzymatically converted into ethylene (**Figures 2**, **3**). Ethylene is a plant growth regulator and stress hormone which plays a key role in causing physiological changes in plants at the molecular level (Pierik et al., 2009). Ethylene interferes with plant growth under salinity stress by inhibiting roots elongation (Glick, 2005), causing defoliation, premature senescence (Shaharoona et al., 2006; Bari and Jones, 2009). To increase resistance against the harmful effects of ethylene, plants are commonly treated with ACC deaminase-producing bacteria (Glick, 2005; Etesami et al., 2015).

So far, many researchers focused on the performance of PGPR with ACC deaminase activity to mitigate the adverse effects of elevated ethylene levels caused by salinity stress (**Table 1**). Siddikee et al. (2010) reported that 25 out of 140 halotolerant bacterial strains isolated from coastal soils of the South Korean Yellow Sea showed ACC deaminase activity. In particular, three of them *Brevibacterium epidermidis*, *Micrococcus yunnanensis*, and *Bacillus aryabhattai* generated more than 40% increase in root elongation and plants’ dry weight when compared to uninoculated salt-stressed canola seedlings (Siddikee et al., 2010). In the study by Kiani et al. (2015), sunflower plants were inoculated with ACC deaminase-producing bacteria, which resulted in better growth in terms of plants’ height and dry weight of shoots and roots. It has also been confirmed that *Achromobacter piechaudii*, an ACC deaminase-containing PGPR, can significantly increase the fresh and dry masses of tomato seedlings (Mayak et al., 2004). The study by Amna et al. (2019) investigated the role of halotolerant ACC deaminase-producing *Bacillus* spp. strains to help wheat seeds germination and seedling growth at different NaCl levels. There are pieces of evidence that ACC deaminase producing PGPRs enhance uptake of essential nutrients like N, P, and K, which consequently increase K^+^/Na^+^ ratios in the stressed plants (Nadeem et al., 2009).

Some fungi use similar mechanisms to alleviate plants stress. Some *Trichoderma* strains produce the enzyme 1-aminocyclopropane-1-carboxylate deaminase that regulates plants’ endogenous 1-aminocyclopropane-1-carboxylic acid (ACC), which is the direct precursor of the plant hormone ethylene. The ACC level regulates the plant’s tolerance to abiotic stress (Zhang et al., 2019). Poveda (2020) showed an impact of the fungi on plant’s hormones as well. The author determined the role that the enzyme chorismate mutase plays in *Trichoderma parareesei* ability to promote tolerance to salinity and drought in plants. This enzyme is at the base of a mechanism that increases the expression of genes related to the hormonal pathways of abscisic acid (ABA) under drought stress, and ethylene (ET) under salt stress.

### Exopolysaccharides Production

The biofilm formation and exopolysaccharide (EPS) production by soil bacteria constitute important strategies to assist metabolism during stress imposed by salinity. EPS produced by PGPRs have a significant impact on plant growth and stress tolerance, such as drought or high salt concentration. They are hydrating compounds that are available for use before the decay of roots or germinating seeds. Bacteria produce polymeric biofilms on a variety of surfaces such as roots and soil, cementing particles, and forming aggregates. This can improve crop performance and soil physicochemical properties (Qurashi and Sabr, 2012; Amna et al., 2019). Salt-tolerant *Halomonas variable* and *Planococcus rifietoensis* strains can improve plant growth and aggregation of soil. These strains showed the formation of a biofilm and accumulated exopolysaccharides as a result of increasing salt stress (Qurashi and Sabr, 2012). Bacterial EPS can help to alleviate salinity stress by reducing the Na^+^ content available for plant uptake (Upadhyay et al., 2011). The kind of EPS produced by *Pseudomonas* spp. (Kasotia et al., 2016), and *Bacillus* spp. (Amna et al., 2019) helped in the binding of free Na^+^ from the soil, thus making Na^+^ unavailable to the soybean and wheat plants, respectively.

### Increased Antioxidant Activity

High soil salinity is also responsible for increased production of reactive oxygen species (ROS) by plants, such as superoxide radical (O_2_^−^), hydrogen peroxide (H_2_O_2_), hydroxyl radical (OH·), and alkaline radicals which have a negative impact on proteins, DNA, lipids, and other biomolecules and cause oxidative effects including plant cell damage and premature senescence or necrosis (Møller et al., 2007; Miller et al., 2010; Habib et al., 2016; Zhang et al., 2018). ROS are produced at a low level in organelles (chloroplasts, mitochondria, peroxisomes) under optimal plant growth conditions. However, under stress conditions, their concentration increases significantly (Miller et al., 2010). A critical system responsible for the production of ROS is the plasma membrane-bound NADPH oxidase (RBOH), which controls cellular redox homeostasis under salinity stress (Hossain and Dietz, 2016; Hossain et al., 2017). Many other plant cell components also play a role in regulating intracellular ROS levels. Among them, the most important are antioxidant enzymes such as peroxidases (POD), catalase (CAT), superoxide dismutase (SOD), ascorbate peroxidase (APX), glutathione reductase (GR), dehydroascorbate reductase (DHAR), monodehydroascorbate reductase (MDAR), glutathione peroxidases (GPX), or glutathione s-transferase (GST) (Yan et al., 2013; Hossain and Dietz, 2016; Sukweenadhi et al., 2018). Non-enzymatic components include glutathione, ascorbic acid, tocopherol, phenolic or polyphenolic compounds (Yan et al., 2013; El-Sayed et al., 2014; Fatma et al., 2014; El-Esawi et al., 2018a). To some species of endophytic fungi or ectomycorrhizal fungi are attributed antioxidant properties that could improve plant’s resistance to plant’s endogenous reactive oxygen species*. Trichoderma* is a genus of soilborne filamentous fungi that comprehend species capable of triggering plants’ defensive mechanisms and inducing tolerance to abiotic stress. Plants’ roots colonised by *T. harzianum* increased the production of antioxidant enzymes (Zehra et al., 2017). Also, Mastouri (2010) showed that plants colonised by *T. harzianum* showed a lower accumulation of lipid peroxides and a higher production of antioxidant compounds such as glutathione, resulting in a mechanism based on the control of the accumulation of reactive oxygen species that occur in stressed plants. *T. harzianum* can accelerate seeds germination while reducing the adverse effects caused to seeds and seedlings by thermal, osmotic, saline, and water stress. Examples of PGPRs and PGPFs, increasing the level of antioxidant enzymes and non-enzymatic redox antioxidants are listed in **Table 1**.

### Biosynthesis of Hydrolytic Enzymes

One of the main indirect mechanisms of plant’s pathogens biocontrol by PGPRs is the production of cell wall degrading enzymes, like chitinases, glucanases, proteases and cellulases that cause lysis of the fungal cell walls (Siddikee et al., 2010; Berrada et al., 2012; Goswami et al., 2014). Teng et al. (2010) reported that halotolerant *Pseudomonas* sp. strain isolated from *Suaeda salsa* is a source of proteinases active against phytopathogenic fungi like *Fusarium oxysporum* (Teng et al., 2010). Two bacterial strains (*B. halotolerans* and *B. pumilus*) isolated from the halophyte *Prosopis strombulifera* were able to produce proteinases inhibiting the growth of *Alternaria* (Sgroy et al., 2009). *B. cereus* and *B. thuringiensis* isolated from salty Tunisian soils were able to produce N-acetyl-β-D-glucosaminidases, chitobiosidases, endochitinases, and they were active against *F. roseum* (Sadfi et al., 2001). The extracellular chitinases of *Serratia marcescens* and *Enterobacter agglomerans* have been indicated as biocontrol agents against *Sclerotium rolfsii*. The ability to suppress *Fusarium oxysporum* and *Rhizoctonia solani* was found in *Peanibacillus* spp., *Bacillus* spp., and S*treptomyces* spp., which synthesise β-1,3-glucanase that are lytic enzymes able to destroy the cell walls of some fungi (Compant et al., 2005; Labuschagne et al., 2010).

### Other Extracellular Molecules

Bacteria secrete many extracellular molecules such as lipo-chitooligosaccharides, bacteriocins, polyamines and volatile organic compounds (**Supplementary Material Table 2**). It has been demonstrated that these molecules often control metabolic pathways and have a role in regulatory functions that increase the plant’s defence and stimulate its growth, stress tolerance, and disease resistance.

Lipo-chitooligosaccharides (LCOs), in particular, are produced by rhizobia and have been found to initiate nodule formation in response to root exudates and flavonoids (Ilangumaran and Smith, 2017). The LCOs molecules have a conserved core and a variable N-Acetyl chain length, with different substitutions (sulfation or glycosylation) and degree of saturation, which account for host specificity (Oldroyd, 2013). Miransari and Smith (2009) reported how *Bradyrhizobium japonicum*, when inoculated in soybean under different salinity levels (from 36 to 61 mM NaCl), enhanced nodulation and growth of plants with the effects becoming more consistent with time.

Rhizobacteria secrete bacteriocins, that are small proteinaceous or peptidic toxins. These act as bactericidal or bacteriostatic agents against competing bacteria and indirectly promote microbial diversity under salinity stress. Some bacteriocins showed a role in plant’s resistance to stress. *Bacillus thuringiensis* is a soybean endosymbiont that, *in vitro*, produces “thuricin 17”, a bacteriocin that is capable of manipulating plant proteome profile, enhancing its tolerance to salinity (Subramanian et al., 2016).

Polyamines (Pas) such as spermidine, spermine, and putrescine, consist of low molecular weight aliphatic amines that can have antioxidant activity. These compounds are present practically in all living organisms and impact reactive oxygen species by scavenging free radicals and inducing the expression of genes related to cellular antioxidant mechanisms. Among all, spermidine which is secreted by *Bacillus megaterium* showed to increase the cellular accumulation of polyamines in plants. The mechanism in *Arabidopsis* involved osmotic stress tolerance *via* the activation of polyamines- mediated cellular signalling, which resulted in greater biomass, higher antioxidant enzyme activity and high photosynthetic capacity in the inoculated plant, compared to the untreated control (Zhou et al., 2016; Ilangumaran and Smith, 2017). Some authors used the definition “systemic induced resistance (SIR)” to cover several bio-protecting mechanisms induced by the PGPRs in plants and that act on multiple functions, once activated at the presence of a pathogenic infection (Numan et al., 2018).

Volatile organic compounds (VOC) are low molecular weight compounds, such as aldehydes, alcohols, ketones and hydrocarbons, which can enter the atmosphere as vapours due to significantly high vapour pressure. They are released from by PGPRs and stimulate plant growth, resulting in increased shoot biomass, and modulated stress responses (Ilangumaran and Smith, 2017). The role of VOCs in the biocontrol of plant’s pathogens and antibiosis is not fully understood, but some of the mechanisms at play gained attention in the last decades and will require further research (Bailly and Weisskopf, 2012). *Paraburkholderia phytofirmans* produced VOCs such as 2-undecanone, 7-hexanol, 3-methylbutanol that stimulate plant growth and induce salinity stress tolerance as demonstrated both *in vitro* and in soil. Growth parameters of *Arabidopsis* plants treated with these VOCs and measured as rosette area, fresh weight, and primary root length were higher than in the control plants (Ledger et al., 2016; Ilangumaran and Smith, 2017). VOCs emitted by *Bacillus subtilis* can stimulate many different hormonal signals in *Arabidopsis thaliana*, which includes cytokinins, salicylic acid, gibberellin, auxin and brassinosteroids (Zhang et al., 2007; Zhang et al., 2008b). Almost 600 genes related to metabolism, auxin homeostasis, cell wall modification and stress response were identified, and these studies showed that VOCs could play an essential role in plant growth and development. PGPRs VOCS can stimulate many chemical and physical changes, some of which could be addressed to improve plants’ tolerance towards abiotic stress (Mantelin and Touraine, 2004; Zhang et al., 2008a).

Several antimicrobial metabolites are the basis of biocontrol mechanisms activated by PGPMs against other microorganisms, and also phytopathogenic species. Strains of *Arthrobacter* spp., *Pseudomonas* spp., *Bacillus* spp., *Streptomyces* spp., can synthesise one or several types of bioactive compounds including amphisin, bacillomycin, 2,4-diacetylphloroglucinol (DAPG), fengycin, hydrogen cyanide, iturin, macrolactin, phenazine-1-carboxylic acid (PCA), pyoluteorin, pyrrolnitrin, surfactin, tensin, tropolone, and viscosinamide (Compant et al., 2005; Hinarejos et al., 2016). However, among bacterial biocontrol agents, the most cited are *Bacillus* spp. (*B. amyloliquefaciens, B. cereus, B. licheniformis, B. pumilus, B. subtilis*) and *Pseudomonas* spp. (*P. chlororaphis, P. fluorescens, P. putida*). Their antagonistic properties against bacterial (*Ralstonia solanacearum, Xanthomonas axonopodis*) and fungal (*F. oxysporum, F. culmorum, P. ultimum, Rhizoctonia solani*) phytopathogens of barley, chickpea, maize, peanut, rice, and wheat were widely proven (Raaijmakers et al., 2002; Yuttavanichakul et al., 2012). Moreover, due to multifaceted mechanisms of action in preventing pathogens’ infections, some strains of *Bacillus* sp. have been commercialized and used for improving crop production (Radhakrishnan et al., 2017; Nakkeeran et al., 2020). The formation of a biofilm around plant’s roots by some *Bacillus* species and the secretion of antagonistic metabolites inhibit pathogenic communities and reduce the occurrence and frequency of the diseases in plants (Radhakrishnan et al., 2017).

One of the antibiosis mechanisms adopted by *Pseudomonas* spp. is the production of hydrogen cyanide. which inhibits the therminal cytochrome c oxidase in the respiratory chain and binds to metalloenzyme (Ramette et al., 2003). However, hydrogen cyanide antagonistic potential against phytopathogens, mainly fungi, is still a matter of discussion (Rijavec and Lapanje, 2016). Ramette et al. (2003) showed a broad spectrum of antifungal activity, whereas Rudrappa et al. (2008) and Blom et al. (2011) reported that hydrogen cyanide is unlikely a biocontrol agent. The authors indicated that pigments and other antibiotic substances are more effective against fungi. Interestingly, Rijavec and Lapanje (2016) proved that HCN regulates phosphate availability of PGPRs and host plants.

## The Interplay Between Halophytes and Their Microbiome: A Glimpse Into the Future

For many years, a wide range of PGPRs and PGPFs have been studied, and some bacterial and fungal isolates, including species of the genera *Pseudomonas, Bacillus, Enterobacter, Klebsiella, Azobacter, Variovorax, Azosprillum*, *Serratia* and *Trichoderma, Aspergillus*, *Penicillium, Phoma* have been used in commercial products (Glick, 2012; Jahagirdar et al., 2019). Nevertheless, the application of PGPRs and PGPFs in the agricultural industry is only a small part of agricultural practice worldwide (Bashan et al., 2014). Disadvantages regarding the utilization of bacteria are connected with properties of the inoculated PGPRs and chiefly depend on their survival in soil, their interaction within indigenous soil microﬂora, and other complex environmental factors (Martinez-Viveros et al., 2010). Moreover, the modes of action of PGPRs are incredibly varied, and not all rhizobacteria have the same effects with identical mechanisms (Choudhary, 2012; Arora et al., 2020). Little is known about PGPFs compared to bacteria regarding their effectiveness in the plant growth-promoting processes. However, many researchers reported beneficial effects of PGPFs application to plants growth by activation of induced systemic resistance (ISR) (Murali et al., 2012; Naziya et al., 2020). Another frontier is the exploitation of interactions between several microorganisms, as it is often from the interaction of several species that the production of bioactive compounds is obtained. Complex interactions between different mycorrhizal species were documented, for instance. Poveda et al. (2019) showed, for example, that plant roots’ colonization by *Trichoderma harzianum* biocontrol strain increases the colonization of the same host by arbuscular mycorrhizal fungal species. The authors, analysing the expression profile of defence-related marker genes, suggested that the phytohormone salicylic acid could play a key role in the modulation of the roots’ colonization process when both fungi are jointly applied.

According to Gangwar et al. (2017) and Egamberdieva et al. (2019), an ideal plant growth-promoting microorganisms (PGPMs) should possess a high rhizosphere competence, enhance plant growth capabilities, have a broad spectrum of action, be safe to the environment, be compatible with other rhizobacteria, and be tolerant to heat, UV radiation, and oxidizing agents. So far, organisms with interesting properties have been isolated, and some possess more than one of the qualities required for a perfect PGPR, however, imagining such an ideal organism capable of accomplishing all the necessary actions, is a kind of extreme. Nevertheless, the direction is the right one because by continuing to search and experimenting, it is possible to find different organisms that together can work with complementary mechanisms. The research on PGPMs as biofertilizers is the most natural and realistic aspiration to face a global agricultural productivity requirement, capable of feeding the world’s population, which is going to escalate to 9 billion people by 2050.

## Author Contributions

All authors contributed to the article and approved the submitted version.

## Funding

This research was conducted in the frame of the Programme “Canaletto, Bilateral Exchange of Scientists Italy-Poland” supported by Polish National Agency for Academic Exchange (project no. PPN/BIL/2018/2/00038/U/00001) and Italian Ministry of Foreign Affairs and International Cooperation (project no. M03375).

## Conflict of Interest

The authors declare that the research was conducted in the absence of any commercial or financial relationships that could be construed as a potential conflict of interest.
