# Supplementary material for: When Salt Meddles Between Plant, Soil, and Microorganisms
Source: Front Plant Sci. 2020 Sep 16;11:553087. doi: 10.3389/fpls.2020.553087 (PMC7525065; doi:10.3389/fpls.2020.553087)
Supplement: Supplementary file 1 [file Table_1.docx]

Supplementary Material

# Supplementary Table 1

**Table 1:** Salinity tolerance of some salt-tolerant plant growth-promoting rhizobacteria (PGPR)

| **Salt-tolerant PGPR** | **Salt tolerance level (mM)** | **References** |
| --- | --- | --- |
| *Bacillus licheniformis* | 400 | Nabti et al. (2013) |
| *Ochrobactrum sp.* | 1000 | Principe et al. (2007) |
| *Rhizobium sp.* | 400 | Thrall et al. (2009) |
| *Pseudomonas protegens* | 1199 | Wang et al. (2015) |
| *Klebsiella sp.* | 1530 | Liu et al. (2014) |
| *Azotobacter sp.* | 200 | Rojas-Tapias et al. (2012) |
| *Enterobacter sp.* | 400 | Yang et al. (2016) |
| *Bacillus sp.* | 2911 | Nielsen et al. (1995) |
| *Bacillus subtilis* | 514 | Abeer et al. (2015) |
| *Rhizobium meliloti* | 500 | Talibart et al. (1994) |
| *Paenibacillus sp.* | 1000 | Ali et al. (2012) |
| *Pseudomonas fluorescens* | 600 | Tewari and Arora (2016) |
| *Mesorhizobium loti* | 700 | Kumar et al. (1999) |
| *Bacillus licheniformis* | 1000 | Goswami et al. (2014) |
| *Serratia marcescens* | 1027 | Singh and Jha (2016) |
| *Azotobacter chroococum* | 1369 | Chaudhary etal. (2013) |
| *Pseudomonas sp.* | 1541 | Sharma et al. (2016) |
| *Pseudomonas aeruginosa* | 1600 | Tewari and Arora (2014b) |
| *Bacillus pumilus* | 2000 | Kannan et al. (2014) |
| *Pseudomonas aeruginosa* | 2000 | Tewari and Arora (2014a) |
| *Zhihengliuella sp.* | 2568 | Jha et al. (2011) |
| *Halobacillus sp.* | 3425 | Ramadoss et al. (2013) |
| *Pseudomonas fluorescens* | 400 | Safari et al. (2016) |
| *Rhizobium leguminosarum* | 343 | Moschetti et al. (2005) |
| *Rhizobium fredii* | 400 | Fujihara and Yoneyama (1993) |
| *Burkholderia phymatum* | 400 | Talbi et al. (2013) |
| *Sinorhizobium medicae* | 513 | Elboutahiri et al. (2010) |
| *Sinorhizobium sp.* | 2100 | Abdelmoumen and El Idrissi (2009) |
| *Serratia marcescens* | 1712 | Nakbanpote et al. (2014) |
| *Synorhizobium meliloti* | 684 | Mrabet et al. (2011) |
| *Rhizobium sp.* | 1000 | Trabelsi et al. (2009) |
| *Halomonas sp.* | 3425 | Mapelli et al. (2013) |
| *Bacillus gibsonii* | 200 | Orhan (2016) |
| *Arthrobacter sp.* | 750 | Kataoka et al. (2017) |
| *Kocuria erythromyxa* | 1712 | Karlidag et al. (2013) |
| *Rhizobium leguminosarum bv*  *phaseoli* | 685 | Abdelmoumen et al. (1999) |
| *Bacillus thuringiensis* | 2000 | Timmusk et al. (2014) |
| *Pseudomonas chlororaphis* | 856 | Egamberdieva et al. (2015) |
| *Pseudomonas sp.* | 1712 | Trung et al. (2016) |

# Supplementary Table 2

**Table 2.** Secondary compounds or enzymes produced by salt-tolerant plant growth-promoting rhizobacteria (PGPR)

| **Secondary compounds or enzymes** | **Salt-tolerant PGPR** | **References** |
| --- | --- | --- |
| **Osmolytes** | | |
| **Glycine betaine** | *Pseudomonas alcaligenes* | Jha et al. (2011) |
|  | *Azospirillum brasilense* | Chowdhury et al. (2007) |
|  | *Bacillus subtilis* | Bremer and Kramer (2000) |
| **Proline** | *Burkholderia* | Barka et al. (2006) |
|  | *Bacillus sp.* | Sziderics et al. (2007) |
|  | *Pseudomonas fluorescens* | Metwali et al. (2015) |
|  | *Pseudomonas strains* | Naz and Bano (2015) |
|  | *Pseudomonas pseudoalcaligenes* | Hanson and Nelson (1978) |
|  | *Azospirillum sp.* | Zarea et al. (2012), Bashan (1999), Casanovas et al. (2003), Bashan and Holguin (1997) |
|  | *Oceanobacillus profundus* | Qurashi and Sabri (2011) |
|  | *Exiguobacterium oxidotolerans* | Bharti et al. (2014) |
|  | *Dietzia natronolimnaea* | Bharti et al. (2016) |
|  | *Pseudomonas koreensis* | Kasotia et al. (2016) |
|  | *Pseudomonas sp.* | Bano and Fatima (2009) |
| **Soluble sugars** | *Pseudomonsa mendocina* | Kohler et al. (2009) |
|  | *Bacillus amyloliquefaciens* | Chen et al. (2016) |
|  | *Azospirillum brasilense* | Bacilio-Jimenez et al. (2001) |
|  | *Serratia marcescens* | Singh and Jha (2016) |
|  | *Bacillus sp.* | Nayer and Reza (2008) |
|  | *Rhizobium tropici* | Figueiredo et al. (2008) |
| **Alanine, serine, threonine, aspartic acid, and other amino**  **acids** | *Pseudomonas fluorescens* | Paul and Lade (2014) |
|  | *Azospirillum braziliense* | Hamdia et al. (2004) |
|  | *Azospirillum lipoferum* | Qudsaia et al. (2013) |
| **Polyamine and amide** | | |
| **Cadaverine** | *Azospirillum brasilense* | Cassan et al. (2009) |
| **N-acetylglutaminylglutamine**  **amide (NAGGA)** | *Pseudomonas putida* | Kets et al. (1996) |
|  | *Pseudomonas syringae* | Kurz et al. (2010) |
| **Exopolysaccharides** | *Rhizobium sp.* | Ahemad and Khan (2012b), Alami et al. (2000) |
|  | *Enterobacter cloacae* | Mahmood et al. (2016) |
|  | *Rhizobium meliloti* | Lloret et al. (1998) |
|  | *Bacillus megaterium* | Nadeem et al. (2016) |
|  | *Bacillus sp.* | Ashraf et al. (2004, 2006) |
|  | *Bacillus tequilensis* | Rolli et al. (2014) |
|  | *Bacillus circulans* | Khodair et al. (2008) |
|  | *Pseudomonas putida* | Sandhya et al. (2009) |
|  | *Zhihengliuella alba* | Siddikee et al. (2011) |
|  | *Bacillus subtilis* | Han and Lee (2005) |
|  | *Proteus penneri* | Naseem and Bano (2014) |
|  | *Pseudomonas sp.* | Singh et al. (1992) |
|  | *Pseudomonas aeruginosa* | Tewari and Arora (2014a, b) |
| **Stress alleviating enzymes** | | |
| **ACC deaminase** | *Pseudomonas sp.* | Nadeem et al. (2007) |
|  | *Achromobacter piechaudii* | Mayak et al. (2004) |
|  | *Pseudomonas fluorescens* | Saravanakumar and Samiyappan (2007) |
|  | *Burkholderia phytofirmans* | Akhtar et al. (2015) |
|  | *Bacillus subtilis* | Abeer et al. (2015) |
|  | *Enterobacter sp.* | Habib et al. (2016) |
|  | *Bacillus halodenitrificans* | Ramadoss et al. (2013) |
|  | *Bacillus licheniformis* | Lim and Kim (2013) |
|  | *Acidovorax sp.* | Esquivel-Cote et al. (2010) |
|  | *Rhizobium sp.* | Ahmad et al. (2013) |
|  | *Pseudomonas stutzeri* | Tank and Saraf (2010) |
|  | *Pseudomonasfluorescens* | Zahir et al. (2009) |
|  | *Enterobacter sp.* | Kim et al. (2014) |
|  | *Serratia quinivirans* | Belimov et al. (2005) |
|  | *Serratia marcescens* | George et al. (2013) |
|  | *Arthrobacter protophormiae* | Barnawal et al. (2014) |
|  | *Pseudomonas koreensis* | Kasotia et al.(2016) |
|  | *Pseudomonas fluorescens* | Nadeem et al. (2016) |
|  | *Bradyrhizobium japonicum* | Shaharoona et al. (2006) |
| **Reactive oxygen species (ROS) scavenging enzymes** | | |
| **Superoxide dismutase** | *Achromobacter xylosoxidans* | Karthikeyan et al. (2012) |
|  | *Serratia marcescens* | Singh and Jha (2016) |
|  | *Pseudomonas putida* | Kang et al. (2014a) |
| **Ascorbate peroxidase** | *Bacillus lentus* | Golpayegani and Tilebeni  (2011) |
|  | *Bacillus megaterium* | Habib et al. (2016) |
|  | *Bacillus lentimorbus* | Nautiyal et al. (2008) |
|  | *Bacillus safensis* | Chakraborty et al. (2013) |
|  | *Burkholderia cepacia* | Kang et al. (2014b) |
|  | *Dietzia natronolimnaea* | Bharti et al. (2016) |
|  | *Pseudomonas stutzeri* | Sharma et al. (2016) |
|  | *Pseudomonas sp.* |  |
| **Catalase** | *Enterobacter sp.* | Habib et al. (2016) |
|  | *Halomonas desiderata* | Bharti et al. (2014) |
|  | *Pseudomonas mendocina* | Kohler et al. (2010) |
|  | *Pseudomonas putida* | Shaik et al. (2011) |

# References of Supplementary Figures

Abdelmoumen, H., and El Idrissi, M.M. (2009). Germination, growth and nodulation of *Trigonella foenum graecum* (Fenu Greek) under salt stress. Afr. J. Biotechnol. 8(11), 2489–2496.

Abdelmoumen, H.A., Filali-Maltout, M., Neyra, A., Belabed, M.M., Idrissi, E. (1999). Effect of high salts concentrations on the growth of rhizobia and responses to added osmotic. J. Appl. Microbiol. 86(6), 889–898.

Abeer, H., Abd Allah, E.F., Alqarawi, A.A., Al-Huqail, A.A., Alshalawi, S.R.M., Wirth, S., Dilfuza, E. (2015). Impact of plant growth promoting *Bacillus subtilis* on growth and physiological parameters of *Bassia indica* (Indian bassia) grown under salt stress. Pak. J. Bot. 47(5), 1735–1741.

Ahemad, M., and Khan, M.S. (2012). Ecological assessment of biotoxicity of pesticides towards plant growth promoting activities of pea (*Pisum sativum*)-specific *Rhizobium sp*. strain MRP1. Emirates J. Food Agric. 24, 334–343.

Ahmad, M., Zahir, Z.A., Nazli, F., Akram, F., Arshad, M., Khalid, M. (2013). Effectiveness of halo-tolerant, auxin producing *Pseudomonas* and *Rhizobium* strains to improve osmotic stress tolerance in mung bean (*Vigna radiata* L.) Braz. J. Microbiol. 44, 1341–1348.

Akhtar, S.S., Andersen, M.N., Naveed, M., Zahir, Z., Liu, F. (2015). Interactive effect of biochar and plant growth-promoting bacterial endophytes on ameliorating salinity stress in maize. Funct. Plant Biol. 42, 770–781.

Alami, Y., Achouak,W., Marol, C., Heulin, T. (2000). Rhizosphere soil aggregation and plant growth promotion of sunflowers by an exopolysaccharide-producing *Rhizobium sp*. strain isolated from sunflower roots. Appl. Environ. Microbiol. 66, 3393–3398.

Ali, S., Charles, T.C., Glick, B.R. (2012). Delay of flower senescence by bacterial endophytes expressing 1-aminocyclopropane-1-carboxylate deaminase. J. Appl. Microbiol. 113, 1139–1144.

Ashraf, M., Hasnain, S., Berge, O. (2006). Effect of exopolysaccharides producing bacterial inoculation on growth of roots of wheat (*Triticum aestivum* L.) plants grown in a salt-affected soil. Int. J. Environ. Sci. Technol. 3, 43–51.

Ashraf, M., Hasnain, S., Berge, O., Mahmood, T. (2004). Inoculating wheat seedlings with exopolysaccharides producing bacteria restricts sodium uptake and stimulates plant growth under salt-stress. Biol. Fertil. Soils 40, 157–162.

Bacilio-Jimenez, M., Aguilar-Flores, S., del Valle, M.V., Prez, A., Zepeda, A., Zenteno, E. (2001). Endophytic bacteria in rice seeds inhibit early colonization of roots by *Azospirillum brasilense*. Soil. Biol. Biochem. 33, 167–172.

Bano, A., and Fatima, M. (2009). Salt tolerance in *Zea mays* (L.) following inoculation with *Rhizobium* and *Pseudomonas*. Biol. Fertil. Soils 45, 405–413.

Barka, A.E., Nowak, J., Clement, C. (2006). Enhancement of chilling resistance of inoculated grape-vine plantlets with a plant growth-promoting rhizobacterium, *Burkholderia phytofirmans* strain PsJN. Appl. Environ. Microbiol. 72, 7246–7252.

Barnawal, D., Bharti, N., Maji, D., Chanotiya, C.S., Kalra, A. (2014). ACC deaminase containing *Arthrobacter protophormiae* induces NaCl stress tolerance through reduced ACC oxidase activity and ethylene production resulting in improved nodulation and mycorrhization in *Pisum sativum*. J. Plant Physiol. 171, 884–894.

Bashan, Y. (1999). Interaction of *Azospirillum spp.* in soils: a review. Biol. Fertil. Soils 29,245–256.

Bashan, Y., and Holguin, G. (1997). *Azospirillum*-plant relationships: environmental and physiological advances (1990–1996). Can. J. Microbiol. 43, 103–121.

Belimov, A.A., Hontzeas, N., Safronova, V.I., Demchinskaya, S.V., Piluzza, G., Bullitta, S., Glick, B.R. (2005). Cadmium-tolerant plant growth-promoting bacteria associated with the roots of Indian mustard (*Brassica juncea* L. Czern.) Soil Biol. Biochem. 37, 241–250.

Bharti, N., Barnawal, D., Awasthi, A., Yadav, A., Kalra, A. (2014). Plant growth promoting rhizobacteria alleviate salinity induced negative effects on growth, oil content and physiological status in *Mentha arvensis*. Acta Physiol. Plant 36, 45–60.

Bharti, N., Pandey, S.S., Barnawal, D., Patel, V.K., Kalra, A. (2016). Plant growth promoting rhizobacteria *Dietzia natronolimnaea* modulates the expression of stress responsive genes providing protection of wheat from salinity stress. Sci. Rep. 6, 34768.

Bremer, E., and Kramer, R. (2000). “Coping with osmotic challenges: osmoregulation through accumulation and release of compatible solutes in bacteria”, in Bacterial stress responses, eds. G. Storz, R. Hengge-Aronis (Washington, DC: ASM Press), 79–97, 501.

Casanovas, E.M., Barassi, C.A., Andrade, F.H., Sueldo, R.J. (2003). *Azospirillum*- inoculated maize plant responses to irrigation restraints imposed during flowering. Cereal Res. Commun. 31, 395–402.

Cassán, F., Perrig, D., Sgroy, V., Masciarelli, O., Penna, C., Luna, V. (2009). *Azospirillum brasilense* Az39 and *Bradyrhizobium japonicum* E109, inoculated singly or in combination, promote seed germination and early seedling growth in corn (*Zea mays* L.) and soybean (*Glycine max* L.). Eur. J. Soil Biol. 45, 28–35.

Chakraborty, U., Chakraborty, B.N., Chakraborty, A.P., Dey, P.L. (2013). Water stress amelioration and plant growth promotion in wheat plants by osmotic stress tolerant bacteria. World J. Microbiol. Biotechnol. 29, 789–803.

Chaudhary, D., Narula, N., Sindhu, S.S., Behl, R.K. (2013). Plant growth stimulation of wheat (*Triticum aestivum* L.) by inoculation of salinity tolerant *Azotobacter* strains. Physiol. Mol. Biol. Plants 19, 515–519.

Chen, L., Liu, Y., Wu, G., Njeri, K.V., Shen, Q., Zhang, N., Zhang, R. (2016). Induced maize salt tolerance by rhizosphere inoculation of *Bacillus amyloliquefaciens* SQR9. Physiol. Plant 158, 34–44.

Chowdhury, S.P., Nagarajan, T., Tripathi, R., Mishra, M.N., Le Rudulier, D., Tripathi, A.K. (2007). Strain-specific salt tolerance and osmoregulatory mechanisms in *Azospirillum brasilense*. FEMS Microbiol. Lett. 267, 72–79.

Egamberdieva, D., Li, L., Lindström, K., Räsänen, L. (2015). A synergistic interaction between salt tolerant *Pseudomonas* and *Mesorhizobium* strains improves growth and symbiotic performance of liquorice (*Glycyrrhiza uralensis* Fish.) under salt stress. Appl. Microbiol. Biotechnol. 100, 2829–2841.

Elboutahiri, N., Thami-Alami, I., Udupa, S.M. (2010). Phenotypic and genetic diversity in *Sinorhizobium meliloti* and *S. medicae* from drought and salt affected regions of Morocco. BMC Microbiol 10, 15.

Esquivel-Cote, R., Ramirez-Gama, R.M., Tsuzuki-Reyes, G., OrozcoSegovia, A., Huante, P. (2010). *Azospirillum lipoferum* strain AZm5 containing 1-aminocyclopropane-1-carboxylic acid deaminase improves early growth of tomato seedlings under nitrogen deficiency. Plant Soil 337(1-2), 65–75.

Figueiredo, M.V.B., Burity, H.A., Martìnez, C.R., Chanway, C.P. (2008). Alleviation of drought stress in the common bean (*Phaseolus vulgaris* L.) by co-inoculation with *Paenibacillus polymyxa* and *Rhizobium tropici*. Appl. Soil Ecol. 40, 182–188.

Fujihara, S., and Yoneyama, T. (1993). Effects of pH and osmotic stress on cellular polyamine contents in the soybean Rhizobia *Rhizobium fredii* P220 and *Bradyrhizobium japonicum* A1017. Appl. Environ. Microbiol. 59(4), 1104–1109.

George, P., Gupta, A., Gopal, M., Thomas, L., Thomas, G.V. (2013). Multifarious beneficial traits and plant growth promoting potential of *Serratia marcescens* KiSII and *Enterobacter sp.* RNF 267 isolated from the rhizosphere of coconut palms (*Cocos nucifera* L.). World J. Microbiol. Biotechnol. 29, 109–117.

Golpayegani, A., Tilebeni, H.G. (2011). Effect of biological fertilizers on biochemical and physiological parameters of Basil (*Ociumum basilicm* L.) medicine plant. Am–Eur. J. Agric. Environ. Sci. 11(3), 411–416.

Goswami, D., Dhandhukia, P., Patel, P., Thakker, J.N. (2014). Screening of PGPR from saline desert of Kutch: Growth promotion in *Arachis hypogea* by *Bacillus licheniformis* A2. Microbiol. Res. 169, 66–75.

Habib, S.H., Kausar, H., Saud, H.M., Ismail, M.R., Othman, R. (2016). Molecular characterization of stress tolerant plant growth promoting rhizobacteria (PGPR) for growth enhancement of rice. Int. J. Agric. Biol. <https://doi.org/10.1155/2016/6284547>

Hamdia, M.B.E., Shaddad, M.A.K., Doaa, M.M. (2004). Mechanisms of salt tolerance and interactive effects of *Azospirillum brasilense* inoculation on maize cultivars grown under salt stress conditions. Plant Growth Regul. 44(2), 165–174.

Han, H.S., Lee, K.D. (2005). Physiological responses of soybean inoculation of *Bradyrhizobium japonicum* with PGPR in saline soil conditions. Res. J. Agric. Biol. Sci. 1, 216–221.

Hanson, A.D., Nelson, C.E. (1978). Betaine accumulation and (14C) formate metabolism in water stressed barley leaves. Plant Physiol. 62, 305–312.

Jha, Y., Subramanian, R.B., Patel, S. (2011). Combination of endophytic and rhizospheric plant growth promoting rhizobacteria in *Oryza sativa* shows higher accumulation of osmoprotectant against saline stress. Acta Physiol. Plant 33, 797–802.

Kang, S.M., Khan, A.L., Waqas, M., You, Y.H., Kim, J.H., Kim, J.G., Hamayun, M., Lee, I.J. (2014b). Plant growth-promoting rhizobacteria reduce adverse effects of salinity and osmotic stress by regulating phytohormones and antioxidants in *Cucumis sativus*. J. Plant. Interact. 9, 673–682.

Kang, S.M., Radhakrishnan, R., Khan, A.L., Kim, M.J., Park, J.M., Kim, B.R. (2014a). Gibberellin secreting rhizobacterium, *Pseudomonas putida* H-2-3 modulates the hormonal and stress physiology of soybean to improve the plant growth under saline and drought conditions. Plant Physiol. Biochem. 84, 115–124.

Kannan, R., Damodaran, T., Pandey, B.K., Umamaheswari, S., Rai, R.B., Jha, S.K., Mishra, V.K., Sharma, D.K., Sah, V. (2014). Isolation and characterization of endophytic plant growth-promoting bacteria (PGPB) associated to the sodicity tolerant polyembryonic mango (*Mangifera indica* L.) root stock and growth vigour in rice under saline sodic environment. Afr. J. Microbiol. Res. 8(7), 628–636.

Karlidag, H., Yildirim, E., Turan, M., Pehluvan, M., Donmez, F. (2013). Plant growth-promoting rhizobacteria mitigate deleterious effects of salt stress on strawberry plants (*Fragaria* *x ananassa*). Hortscience. 48(5), 563–567.

Karthikeyan, B., Joe, M.M., Islam, M.R., Sa, T. (2012). ACC deaminase containing diazotrophic endophytic bacteria ameliorate salt stress in *Catharanthus roseus* through reduced ethylene levels and induction of antioxidative defense systems. Symbiosis 56, 77–86.

Kasotia, A., Varma, A., Tuteja, N., Choudhary, D.K. (2016). Amelioration of soybean plant from saline induced condition by exopolysaccharide producing *Pseudomonas*-mediated expression of high affinity K+ transporter (HKT1) gene. Curr. Sci. 111(12), 25.

Kataoka, R., Güneri, E., Turgay, O.C., Yaprak, A.E., Sevilir, B., Başköse, I. (2017). Sodium-resistant plant growth-promoting rhizobacteria isolated from a halophyte, *Salsola grandis*, in saline-alkaline soils of Turkey. Eur. J. Soil Sci. 6(3), 216–225.

Kets, E.P.W., de Bont, J.A.M., Heipieper, H.J. (1996). Physiological response of *Pseudomonas putida* S12 subjected to reduced water activity. FEMS Microbiol. Lett. 139, 133–137.

Khodair, T.A., Galal, G.F., El-Tayeb, T.S. (2008). Effect of inoculating wheat seedlings with exopolysaccharide- producing bacteria in saline soil. J. Appl. Sci. Res. 4, 2065–2070.

Kim K, Jang YJ, Lee SM, Oh BT, Chae JC, Lee KJ (2014) Alleviation of salt stress by *Enterobacter* sp. EJ01 in tomato and *Arabidopsis* is accompanied by up-regulation of conserved salinity responsive factors in plants. Mol Cell 37:109–117

Kohler, J., Caravaca, F., Roldan, A. (2010). An AM fungus and a PGPR intensify the adverse effects of salinity on the stability of rhizosphere soil aggregates of *Lactuca sativa*. Soil Biol. Biochem. 42, 429–434.

Kohler, J., Hernández, J.A., Caravaca, F., Roldan, A. (2009). Induction of antioxidant enzymes is involved in the greater effectiveness of a PGPR versus AM fungi with respect to increasing the tolerance of lettuce to severe salt stress. Environ. Exp. Bot. 65, 245–252.

Kumar, H., Arora, N.K., Kumar, V., Maheshwari, D.K. (1999). Isolation, characterization and selection of salt tolerant rhizobia nodulating *Acacia catechu* and *A. nilotica*. Symbiosis 26, 279–288.

Kurz, M., Burch, A.Y., Seip, B., Lindow, S.E., Gross, H. (2010). Genome-driven investigation of compatible solute biosynthesis pathways of *Pseudomonas syringae pv. syringae* and their contribution to water stress tolerance. Appl. Environ. Microbiol. 76(16), 5452–5462.

Lim, S.J., Kim, S.D. (2013). Induction of drought stress resistance by multi-functional PGPR *Bacillus licheniformis* K11 in pepper. Plant Pathol. J. 29(2), 201–208.

Liu, W., Hou, J., Wang, Q., Dinga, L., Luo, Y. (2014). Isolation and characterization of plant growth-promoting rhizobacteria and their effects on phytoremediation of petroleum-contaminated saline-alkali soil. Chemosphere 117C (1), 303–308.

Lloret, J., Wulff, B.B.H., Rubio, J.M., Downie, J.A., Bonilla, I., Rivilla, R. (1998). Exopolysaccharide II production is regulated by salt in the halotolerant strain *Rhizobium meliloti* EFB1. Appl. Environ. Microbiol. 64, 1024–1028.

Mahmood, S., Daur, I., Al-Solaimani, S.G., Ahmad, S., Madkour, M.H., Yasir, M., Hirt, H., Ali, S., Ali, Z. (2016). Plant growth promoting rhizobacteria and silicon synergistically enhance salinity tolerance of mung bean. Front. Plant. Sci. 7, 876.

Mapelli, F., Marasco, R., Rolli, E., Barbato, M., Cherif, H., Guesmi, A., Ouzari, I., Daffonchio, D., Borin, S. (2013). Potential for plant growth promotion of rhizobacteria associated with *Salicornia* growing in Tunisian hypersaline soils. Biomed. Res. Int. 2013 (248078):13. https://doi.Org/10.1155/2013/248078

Mayak, S., Tirosh, T., Glick, B.R. (2004). Plant growth promoting bacteria confer resistance in tomato plants to salt stress. Plant Physiol. Biochem. 42, 565–572.

Metwali, E.M., Abdelmoneim, T.S., Bakheit, M.A., Kadasa, N.M. (2015). Alleviation of salinity stress in faba bean (*Vicia faba* L.) plants by inoculation with plant growth promoting rhizobacteria (PGPR). Plant Omics 8(5), 449.

Moschetti, G., Peluso, A., Protopapa, A., Anastasio, M., Pepe, O., Defez, R. (2005). Use of nodulation pattern, stress tolerance, *nod*C gene amplification, RAPD-PCR and RFLP-16S rDNA to discriminate genotypes of *Rhizobium leguminosarum* biovar *viciae*. Syst. Appl. Microbiol. 28, 619–631.

Mrabet, M., Abdellatif, E., Zribi, K., Mhamdi, R., Djébali, N. (2011). *Sinorhizobium meliloti* can protect *Medicago truncatula* from infection by *Phoma medicaginis*. Phytopathol. Mediterr. 50, 183–191.

Nabti, E.H., Mokrane, N., Ghoul, M., Manyani, H., Dary, M., Megias, M.G. (2013). Isolation and characterization of two halophilic *Bacillus* (*B. licheniformis* and *Bacillus sp*) with antifungal activity. J. Ecol. Heal. Environ. 1(1), 13–17.

Nadeem, S.M., Ahmad, M., Naveed, M., Imran, M., Zahir, Z.A., Crowley, D.E. (2016). Relationship between in vitro characterization and comparative efficacy of plant growth-promoting rhizobacteria for improving cucumber salt tolerance. Arch Microbiol 198(4), 379–387.

Nadeem, S.M., Zahir, Z.A., Naveed, M., Arshad, M. (2007). Preliminary investigations on inducing salt tolerance in maize through inoculation with rhizobacteria containing ACC-deaminase activity. Can. J. Microbiol. 53, 1141–1149.

Nakbanpote, W., Panitlurtumpai, N., Sangdee, A., Sakulpone, N., Sirisom, P., Pimthong, A. (2014). Salt-tolerant and plant growth-promoting bacteria isolated from Zn/Cd contaminated soil: identification and effect on rice under saline conditions. J. Plant Interact. 9(1), 379–387.

Naseem, H., and Bano, A. (2014). Role of plant growth-promoting rhizobacteria and their exopolysaccharide in drought tolerance of maize. J. Plant Interact 9(1), 689–701.

Nautiyal, C., Govindarajan, R., Lavania, M., Pushpangadan, P. (2008). Novel mechanism of modulating natural antioxidants in functional foods: involvement of plant growth promoting rhizobacteria NRRL B-30488. J. Agric, Food Chem, 56, 4474–4481.

Nayer, M., Reza, H. (2008). Drought-induced accumulation of soluble sugars and proline in two maize varieties. World Appl. Sci. J. 3, 448–453.

Naz, R., and Bano, A. (2015). Molecular and physiological responses of sunflower (*Helianthus annuus* L.) to PGPR and SA under salt stress. Pak. J. Bot. 47(1), 35–42.

Nielsen, P., Fritze, D., Priest, F. (1995). Phenetic diversity of alkaliphilic *Bacillus* strains: proposal for nine new species. Microbiology 141, 1745–1761.

Orhan, F. (2016). Alleviation of salt stress by halotolerant and halophilic plant growth-promoting bacteria in wheat (*Triticum aestivum*). Braz. J. Microbiol. 47(3), 621–627.

Paul, D., and Lade, H. (2014). Plant-growth-promoting rhizobacteria to improve crop growth in saline soils: a review. Agron. Sustain. Dev. 34, 737–752.

Principe, A., Alvarez, F., Castro, M.G., Zachi, L., Fischer, S.E., Mori, G.B., Jofre, E. (2007). Biocontrol and PGPR features in native strains isolated from saline soils of Argentina. Curr. Microbiol. 55, 314–322.

Qudsaia, B., Noshinil, Y., Asghari, B., Nadia, Z., Abida, A., Fayazul, H. (2013). Effect of *Azospirillum* inoculation on maize (*Zea mays* L.) under drought stress. Pak. J. Bot. 45, 13–20.

Qurashi, A.W., and Sabri, S.N. (2011). Osmoadaptation and plant growth promotion by salt tolerant bacteria under salt stress. Afr. J. Microbiol. Res. 5(2), 3546–3554.

Ramadoss, D., Lakkineni, V.K., Bose, P., Ali. S., Annapurna, K. (2013). Mitigation of salt stress in wheat seedlings by halotolerant bacteria isolated from saline habitats. Springer Plus 2, 1–7.

Rojas-Tapias, D., Moreno-Galván, A., Pardo-Díaz, S., Obando, M., Rivera, D., Bonilla, R. (2012). Effect of inoculation with plant growth-promoting bacteria (PGPB) on amelioration of saline stress in maize (*Zea mays*). Appl. Soil Ecol. 61, 264–272.

Rolli, E., Marasco, R., Vigani, G., Ettoumi, B., Mapelli, F., Deangelis, M.L. (2014). Improved plant resistance to drought is promoted by the root-associated microbiome as a water stress-dependent trait. Environ. Microbiol. 17, 316–331.

Safari, D., Jamali, F., Nooryazdan, H.R., Bayat, F. (2016). Screening fluorescent pseudomonads isolated from wheat rhizosphere for plant growth- promoting and salt tolerance properties. Biol. Forum– Int. J. 8(1), 35–42.

Sandhya, V., Ali, S.K.Z., Minakshi, G., Reddy, G., Venkateswarlu, B. (2009). Alleviation of drought stress effects in sunflower seedlings by the exopolysaccharides producing *Pseudomonas putida* strain GAP-P45. Biol. Fertil. Soils 46, 17–26.

Saravanakumar, D., Samiyappan, R. (2007). ACC deaminase from *Pseudomonas fluorescens* mediated saline resistance in groundnut (*Arachis hypogea*) plants. J. Appl. Microbiol. 10, 1283–1292

Shaharoona, B., Arshad, M., Zahir, Z.A. (2006). Effect of plant growth promoting rhizobacteria containing ACC-deaminase on maize (*Zea mays* L.) growth under axenic conditions and on nodulation in mung bean (*Vigna radiata* L.) Lett. Appl. Microbiol. 42, 155-159.

Shaik, Z.A., Vardharajula, S., Minakshi, G., Venkateswar, R.L., Bandi, V. (2011). Effect of inoculation with a thermotolerant plant growth promoting *Pseudomonas putida* strain AKMP7 on growth of wheat (*Triticum spp.*) under heat stress. J. Plant Interact. 6, 239–246.

Sharma, S., Kulkarni, J., Jha, B. (2016). Halotolerant rhizobacteria promote growth and enhance salinity tolerance in peanut. Front. Microbiol. 7, 1600.

Siddikee, M.A., Glick, B.R., Chauhan, P.S., Yim, W.J., Sa, T. (2011). Enhancement of growth and salt tolerance of red pepper seedlings (*Capsicum annuum* L.) by regulating stress ethylene synthesis with halotolerant bacteria containing 1-aminocyclopropane-1-carboxylic acid deaminase activity. Plant Physiol. Biochem. 49, 427–443.

Singh, R.P., and Jha, P.N. (2016). The multifarious PGPR Serratia marcescens CDP-13 augments induced systemic resistance and enhanced salinity tolerance of wheat (*Triticum aestivum* L.) PLoS One 11(6):e0155026

Singh, S., Koehler, B., Fett, W.F. (1992). Effect of osmolarity and dehydration on alginate production by fluorescent pseudomonads. Curr. Microbiol. 25, 335–339.

Sziderics, A.H., Rasche, F., Trognitz, F., Sessitsch, A., Wilhelm, E. (2007). Bacterial endophytes contribute to abiotic stress adaptation in pepper plants (*Capsicum annuum* L.). Can. J. Microbiol. 53, 1195–1202.

Talbi, C., Argandoña, M., Salvador, M., Alché, J.D., Vargas, C., Bedmar, E.J., Delgado, M.J. (2013). *Burkholderia phymatum* improves salt tolerance of symbiotic nitrogen fixation in *Phaseolus vulgaris*. Plant Soil 367, 673–685.

Talibart, R., Jebbar, M., Gouesbet, G. (1994). Osmoadaptation in rhizobia: ectoine-induced salt tolerance. J. Bacteriol. 176, 5210–5217.

Tank, N., and Saraf, M. (2010). Salinity-resistant plant growth promoting rhizobacteria ameliorates sodium chloride stress on tomato plants. J. Plant Interact. 5, 51–58.

Tewari, S., Arora, N.K. (2014a). Multifunctional exopolysacccharides from *Pseudomonas aeruginosa* PF23 involved in plant growth stimulation, biocontrol and stress amelioration in sunflower under stress conditions. Curr. Microbiol. 69, 484–494.

Tewari, S., Arora, N.K. (2014b). Talc based exopolysaccharides formulation enhancing growth and production of *Helianthus annuus* under saline conditions. Cell Mol. Biol. 60(5), 73–81.

Tewari, S., and Arora, N.K. (2016). Fluorescent *Pseudomonas sp.* PF17 as an efficient plant growth regulator and biocontrol agent for sunflower crop under saline conditions. Symbiosis. https://doi.org/10.1007/s13199-016-0389-8

Thrall, P.H., Broadhurst, L.M., Hoque, M.S., Bagnall, D.J. (2009). Diversity and salt tolerance of native Acacia rhizobia isolated from saline and non-saline soils. Aust. Ecol. 34, 950–963.

Timmusk, S., Abd El-Daim, I., Copolovici, L., Tanilas, T., Kännaste, A., Behers, L., Nevo, E., Seisenbaeva, G., Stenström, E., Niinemets, U. (2014). Drought-tolerance of wheat improved by rhizosphere bacteria from harsh environments: enhanced biomass production and reduced emissions of stress volatiles. PLoS One 9:e96086. <https://doi.org/10.1371/journal.pone.0096086>

Trabelsi, D., Mengoni, A., Aouani, M.E., Bazzicalupo, M. (2009). Genetic diversity and salt tolerance of bacterial communities from two Tunisian soils. Ann. Microbiol. 59, 1–8.

Trung, N.T., Hieu, H.V., Thuan, N.H. (2016). Screening of strong 1-aminocyclopropane-1-carboxylate deaminase producing bacteria for improving the salinity tolerance of cowpea. Appl. Micro Open Access 2:1000111. https://doi.org/10.4172/2471-9315.1000111

Wang, X., Mavrodi, D.V., Ke, L., Mavrodi, O.V., Yang, M., Thomashow, L.S. (2015). Biocontrol and plant growth-promoting activity of rhizobacteria from Chinese fields with contaminated soils. Microb. Biotechnol. 8, 404–418.

Yang, H., Hu, J., Long, X., Liu, Z., Rengel, Z. (2016). Salinity altered root distribution and increased diversity of bacterial communities in the rhizosphere soil of Jerusalem artichoke. Sci. Rep. 6, 2.

Zahir, A.Z., Ghani, U., Naveed, M., Nadeem, S.M., Asghar, H.N. (2009). Comparative effectiveness of *Pseudomonas* and *Serratia sp.* containing ACC deaminase for improving growth and yield of wheat (*Triticum aestivum* L.) under salt stressed conditions. Arch. Microbiol. 191, 415–424.

Zarea, M.J., Hajinia, K., Goltapeh, E.M., Rejali, F., Varma, A. (2012). Effect of *Piriformospora indica* and *Azospirillum* strains from saline or non-saline soil on mitigation of the effects of NaCl. Soil Biol. Biochem. 45, 139–146.
